# Identification of Immune and Viral Correlates of Norovirus Protective Immunity through Comparative Study of Intra-Cluster Norovirus Strains

**DOI:** 10.1371/journal.ppat.1003592

**Published:** 2013-09-05

**Authors:** Shu Zhu, Doron Regev, Makiko Watanabe, Danielle Hickman, Nissin Moussatche, Desyree Murta Jesus, Shannon M. Kahan, Sawsan Napthine, Ian Brierley, Robert N. Hunter, Divya Devabhaktuni, Melissa K. Jones, Stephanie M. Karst

**Affiliations:** 1 College of Medicine, Department of Molecular Genetics and Microbiology, University of Florida, Gainesville, Florida, United States of America; 2 Division of Virology, Department of Pathology, University of Cambridge, Cambridge, United Kingdom; University of North Carolina at Chapel Hill, United States of America

## Abstract

Whether or not primary norovirus infections induce protective immunity has become a controversial issue, potentially confounded by the comparison of data from genetically distinct norovirus strains. Early human volunteer studies performed with a norovirus-positive inoculum initially led to the conclusion that primary infection does not generate long-term, protective immunity. More recently though, the epidemiological pattern of norovirus pandemics has led to the extrapolation that primary norovirus infection induces herd immunity. While these are seemingly discordant observations, they may in fact reflect virus strain-, cluster-, or genogroup-specific differences in protective immunity induction. Here, we report that highly genetically related intra-cluster murine norovirus strains differ dramatically in their ability to induce a protective immune response: Primary MNV-3 infection induced robust and cross-reactive protection, whereas primary MNV-1 infection induced modest homotypic and no heterotypic protection. In addition to this fundamental observation that intra-cluster norovirus strains display remarkable differences in protective immunity induction, we report three additional important observations relevant to norovirus:host interactions. First, antibody and CD4^+^ T cells are essential to controlling secondary norovirus infections. Second, the viral minor structural protein VP2 regulates the maturation of antigen presenting cells and protective immunity induction in a virus strain-specific manner, pointing to a mechanism by which MNV-1 may prevent the stimulation of memory immune responses. Third, VF1-mediated regulation of cytokine induction also correlates with protective immunity induction. Thus, two highly genetically-related norovirus strains displayed striking differences in induction of protective immune responses, strongly suggesting that the interpretation of norovirus immunity and vaccine studies must consider potential virus strain-specific effects. Moreover, we have identified immune (antibody and CD4^+^ T cells) and viral (VP2 and possibly VF1) correlates of norovirus protective immunity. These findings have significant implications for our understanding of norovirus immunity during primary infections as well as the development of new norovirus vaccines.

## Author Summary

Human noroviruses are a significant cause of gastroenteritis outbreaks worldwide and likely the leading cause of severe childhood diarrhea. An efficacious norovirus vaccine would have a major impact on human health but will undoubtedly be confounded by several roadblocks. First, the norovirus genus is highly genetically, and potentially antigenically, diverse. Second, it is currently unclear whether human noroviruses elicit lasting protective immunity upon natural infection. Here, we test the hypothesis that noroviruses display virus strain-specific differences in their stimulation of protective immunity. Indeed, our results reveal that two highly genetically related murine norovirus strains differ dramatically in their stimulation of protective immune responses. Moreover, we demonstrate that antibody and CD4^+^ T cells are absolutely essential to protecting from a secondary norovirus infection. Finally, we have revealed two viral correlates of protective immunity induction – VF1-mediated cytokine antagonism and VP2-dependent inhibition of antigen presenting cell maturation. Collectively, this information not only offers a potential explanation for the seemingly discordant results regarding human norovirus protective immunity but it also brings to light a previously unrecognized complexity in developing an efficacious human norovirus vaccine – individual virus strains may differ significantly in their interactions with the host immune system and thus in their immunogenicity.

## Introduction

Noroviruses (NoVs) represent a genus within the *Caliciviridae* family of viruses, comprised of non-enveloped positive-sense RNA viruses. The NoV genome is 7.4 to 7.7 kb in length typically organized into three open reading frames (ORF1-3), with the 5′ proximal ORF1 encoding a large polyprotein cleaved into six mature nonstructural proteins; ORF2 encoding the major capsid protein referred to as VP1; and ORF3 encoding a minor structural protein referred to as VP2 [1]–[4]. A fourth ORF present only in murine NoV (MNV) genomes has recently been shown to produce a protein called virulence factor 1 (VF1) that regulates the innate immune response [5]. Human noroviruses (HuNoVs) are a major cause of gastroenteritis outbreaks worldwide, implicated in over 95% of non-bacterial outbreaks. These highly infectious and ubiquitous viruses spread person-to-person and via fecal-oral contamination, and symptomatically infect people of all ages [6], [7]. They are also now recognized to be an important cause of sporadic diarrheal disease. In fact, emerging evidence indicates that HuNoVs are now the leading cause of severe childhood gastroenteritis at least in the United States [8], [9], supplanting rotaviruses since the introduction of an effective rotavirus vaccine. One literature review of the association of HuNoVs with severe diarrhea concluded that HuNoVs likely cause over 1 million hospitalizations and 200,000 deaths in children annually [10].

The NoV genus is currently divided into five genogroups (GI-GV) and further divided into approximately 30 genotypes or clusters (e.g., GI.1 refers to a genogroup 1, genotype/cluster 1 NoV strain), based on VP1 protein sequence similarity [11]–[14]. Members of the GI and GII genogroups are the most prevalent in human infections, with GII.4 strains being responsible for 70–80% of HuNoV outbreaks worldwide since 2002 [15], [16]. This is underscored by the global dominance of a recently emergent GII.4 variant called the Sydney strain [17], [18]. The NoV genus displays extreme genetic diversity [13]. For example, pandemic GII.4 HuNoV strains (intra-cluster variants) differ by 5–7% in their VP1 amino acid sequences whereas they differ by 37–38% compared to the prototype GI.1/Norwalk strain (inter-genogroup variants). This genetic and potentially antigenic variation is likely to pose a major obstacle to HuNoV vaccine development, underscored by the lack of inter-genogroup cross-protection recently reported in a chimpanzee model of HuNoV infection [19].

However, NoV protective immunity appears to be more complex than can be explained solely by antigenic variability: Human volunteer challenge studies performed with a GI.1 HuNoV clearly demonstrated that a proportion of individuals are repeatedly susceptible to homologous HuNoV re-challenge [20]–[22]. These studies further suggest that people develop short-term, but not long-term, HuNoV protective immunity [21]. In contrast to these early human volunteer studies, more recent studies of the prevalent GII.4 HuNoVs support the development of herd immunity underlying their epochal pattern of evolution [23]–[25]. Because NoVs are so genetically diverse, it is possible that these discrepant observations are due to HuNoV strain-specific differences in the stimulation of memory immune responses. Alternatively, it is possible that some or all NoVs elicit short-term, but not long-term, protection which is of sufficient duration to drive the emergence of antigenically distinct viruses over a relatively short time period. These possibilities are not mutually exclusive. This information is critical to the design of an effective NoV vaccination program which will undoubtedly require frequent re-formulations as novel pandemic strains emerge, similar to influenza virus vaccination.

Unraveling the complexities of HuNoV protective immunity is challenging for several reasons. First, HuNoVs are highly prevalent on a global scale which means that nearly everyone has been previously exposed to one or more HuNoV strains in their lifetime [26]. This common exposure history represents a major hurdle to the interpretation of any HuNoV immunity study. Second, they are fairly species-specific and do not appear to infect small animals, although large animal models are in development [19], [27]–[29]. An important model system that is shedding light on NoV pathogenesis, immunity, and replication strategies is the murine model of NoV infection based on infecting mice with murine NoVs (MNVs) [30]. Numerous MNV strains have been reported from diverse geographical locations. Several studies have highlighted phenotypic variation among these strains including differences in clearance rates from infected mice, cytopathicity profiles in cultured cells, and glycan binding profiles [31]–[34]. We previously reported that two genetically related MNV strains display distinct in vivo properties, with MNV-3 being attenuated relative to MNV-1 in both wild-type and STAT1^−/−^ mice; this attenuation is not due to decreased in vivo replication since MNV-3 can reach higher peak intestinal titers than MNV-1 [35]. It has also been reported that MNV-3 establishes a more prolonged infection than MNV-1 [32], [36].

Regarding the utility of the murine model to study NoV protective immunity, it is advantageous because exposure events can be carefully controlled. Moreover, challenge studies can be performed in a variety of mouse strains genetically deficient in specific components of the immune response to probe host determinants of protection. Our previous studies have demonstrated that high-dose MNV-1 infection fails to elicit robust protection as defined by measuring fecal inconsistency as a marker of disease and reductions in virus loads [37], and that the weak memory immune responses elicited by a primary MNV-1 exposure further wane over time [7]. In contrast, Chachu et al. demonstrated that multiple exposures to MNV-1 do elicit a lasting protective immune response that involves B cells, CD4^+^ T cells, and CD8^+^ T cells in a tissue-specific manner [38]. Collectively, these results suggest that primary infection with a MNV elicits suboptimal immunity whereas multiple exposures are capable of boosting immunity. Importantly, these two sets of studies address distinct questions – the first elucidating the host immune response to a primary NoV infection mimicking natural infection and the second addressing the response to a prime:boost vaccination regiment. Both of these topics are fundamentally important to understanding how people will ultimately respond to NoV vaccines in the face of pre-exposures to natural virus infection, warranting independent investigation.

Our studies described here directly test whether NoV strains differ in their capacity to elicit protective immunity upon a primary infection. To this end, we compared the magnitude of the memory immune response elicited by genetically related but pathologically distinct MNV strains, namely MNV-1 and MNV-3. Indeed, we report here that the more attenuated but persistent MNV-3 elicits remarkably more robust protection than the more virulent but acute MNV-1. Moreover, this protection is cross-reactive towards MNV-1. We also show that, while type I interferon (IFN), type II IFN, and CD8^+^ T cells are not critical for MNV-3 protection, antibody and CD4^+^ T cells are essential. Finally, our studies reveal that the minor structural protein VP2 is partially responsible for dictating NoV protective immunity, a phenotype that correlates with its ability to regulate antigen presenting cell (APC) maturation. In addition to APC maturation, MNV-1 and MNV-3 differ in their ability to antagonize cytokine induction likely via the newly described VF1 [5]. Collectively, these results point to a multifactorial antagonism strategy used by MNV-1 to avoid stimulating memory immune responses. MNV-1 and MNV-3 differ by only 5% in their VP1 protein sequences, identical to the level of similarity between pandemic GII.4 HuNoV strain variants. The implication regarding HuNoV infections is that there may be not only inter-genogroup, but also intra-genogroup and even intra-cluster, variations in protective immunity induction and possibly vaccine efficacy. Thus, in addition to a lack of heterotypic protection [19], HuNoV strains may also differ remarkably in their ability to induce homotypic protective immune responses.

## Results

### MNV-3, but not MNV-1, elicits robust homotypic and heterotypic protective immunity

**Figure 1:**
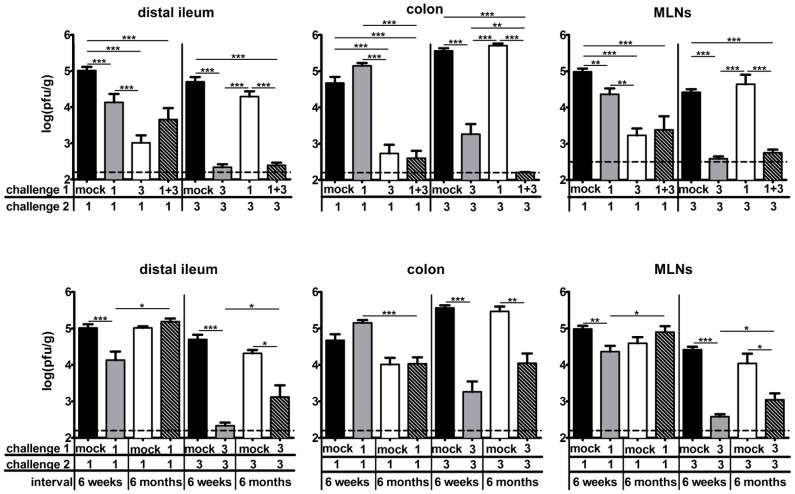
A relatively attenuated MNV strain induces more robust homotypic and heterotypic immunity than a virulent strain. **A**) Groups of wild-type B6 mice (a minimum of 5 mice per group tested in two independent experiments) were inoculated with mock inoculum, 10^4^ TCID_50_ units of MNV-1 (1), 10^4^ TCID_50_ units of MNV-3 (3), or 5×10^3^ TCID_50_ units of each MNV-1 and MNV-3 (1+3) by the peroral route; this is referred to as challenge 1. Six weeks later, mice were infected with 10^7^ TCID_50_ units of MNV-1 or MNV-3; this is referred to challenge 2. One day following challenge 2, animals were perfused, the indicated organs harvested, and viral burden determined by plaque assay. The data for all mice in each group were averaged. Mice receiving mock inoculum at challenge 1 and virus infection at challenge 2 (the primary infection groups) are indicated by black bars; mice receiving homologous virus at challenges 1 and 2 are indicated by grey bars; mice receiving heterologous virus at challenges 1 and 2 are indicated by white bars; and mice co-infected with MNV-1 and MNV-3 at challenge 1 and either MNV-1 or MNV-3 at challenge 2 are indicated by black striped bars. Limits of detection are indicated by dashed lines. **B**) Homotypic challenge studies were performed on groups of B6 mice (n = 4) as described in panel A, with the exception that challenge 2 was administered to the mice six months (instead of six weeks) following challenge 1. The parallel groups of mice challenged after six weeks from panel A are included here for clarity. In all experiments, the indicated groups were compared in statistical analysis as described in the Methods. MLNs = mesenteric lymph nodes.

To test whether intra-cluster NoV strains differ in protective immunity induction, we compared the magnitude of protection elicited by MNV-1 and MNV-3. We used reductions in virus loads during a secondary challenge compared to primary infection as a measure of protection since MNV-3 is avirulent in wild-type mice [35]. Virus loads were measured at 1 day post-challenge because this is the time point at which peak intestinal titers are observed during primary infections [35]. Specifically, mice were inoculated with mock inoculum or 10^4^ TCID_50_ units of MNV and then challenged with 10^7^ TCID_50_ units of homologous virus six weeks later. One day post-challenge, three tissues were collected for virus load determination – the distal ileum as representative of the small intestine, the colon as representative of the large intestine, and mesenteric lymph nodes (MLNs) that drain the intestine. Under these conditions, MNV-1 and MNV-3 reached comparable titers during primary infection, although primary MNV-3 colonic titers were generally higher than MNV-1 titers (**Figure 1** **; black bars**). In control studies in which mice received MNV-1 or MNV-3 at primary infection and mock inoculum at secondary challenge, no virus was detected in any tissue (data not shown). Consistent with our previously published results of weak MNV-1 protection in both 129SvEv and B6 mice [37], prior MNV-1 exposure resulted in only modest reductions in secondary titers in the distal ileum and MLNs upon a homotypic challenge; no protection was observed in the colon (**Figure 1A** **, grey bars**). In striking contrast, MNV-3 induced robust homotypic protection indicated by nearly undetectable levels of virus during secondary challenge in the distal ileum and MLNs and a significant reduction in colonic titers. We also tested whether MNV-1 and MNV-3 elicited heterotypic protection by performing primary infections with one strain and secondary challenges with the reciprocal strain (**Figure 1A** **; white bars**). Remarkably, primary MNV-3 infection induced significantly better protection to a MNV-1 challenge than MNV-1 itself in all tissues. In contrast, primary MNV-1 infection afforded only modest distal ileum protection to a MNV-3 challenge and no protection in the colon or MLNs. This led us to question whether MNV-1 actively suppresses the induction of memory immune responses. To test this, we co-infected mice with equivalent doses of MNV-1 and MNV-3 and challenged with each virus strain separately. In this setting, MNV-3 was still able to mount a protective response to MNV-1 and MNV-3 re-challenges (**Figure 1A** **; black striped bars**). The magnitude of protection elicited by MNV-1+MNV-3 co-infection was statistically similar to singular MNV-3 infection in all instances except for the colon in MNV-3-rechallenged mice; in this case, the co-infection actually elicited more robust protection compared to MNV-3 alone. These data argue against an active MNV-1 suppression mechanism. We have previously reported that the weak homotypic protective immunity elicited by MNV-1 further wanes over time [7]. To determine whether the more robust MNV-3 protective immunity is long-lived, we performed homotypic MNV-1 and MNV-3 challenges with a 6-month interval between primary and secondary infections. The magnitude of MNV-1 and MNV-3 homotypic protective immunity with a 6-month interval was reduced compared to that observed in a 6-week challenge (**Figure 1B**), suggesting that even robust NoV protective immunity wanes over time. Overall, a more attenuated MNV strain elicits robust homotypic and heterotypic protection whereas a more virulent strain does not.

### MNV-3 elicits protection from disease independent of type I interferon

**Figure 2:**
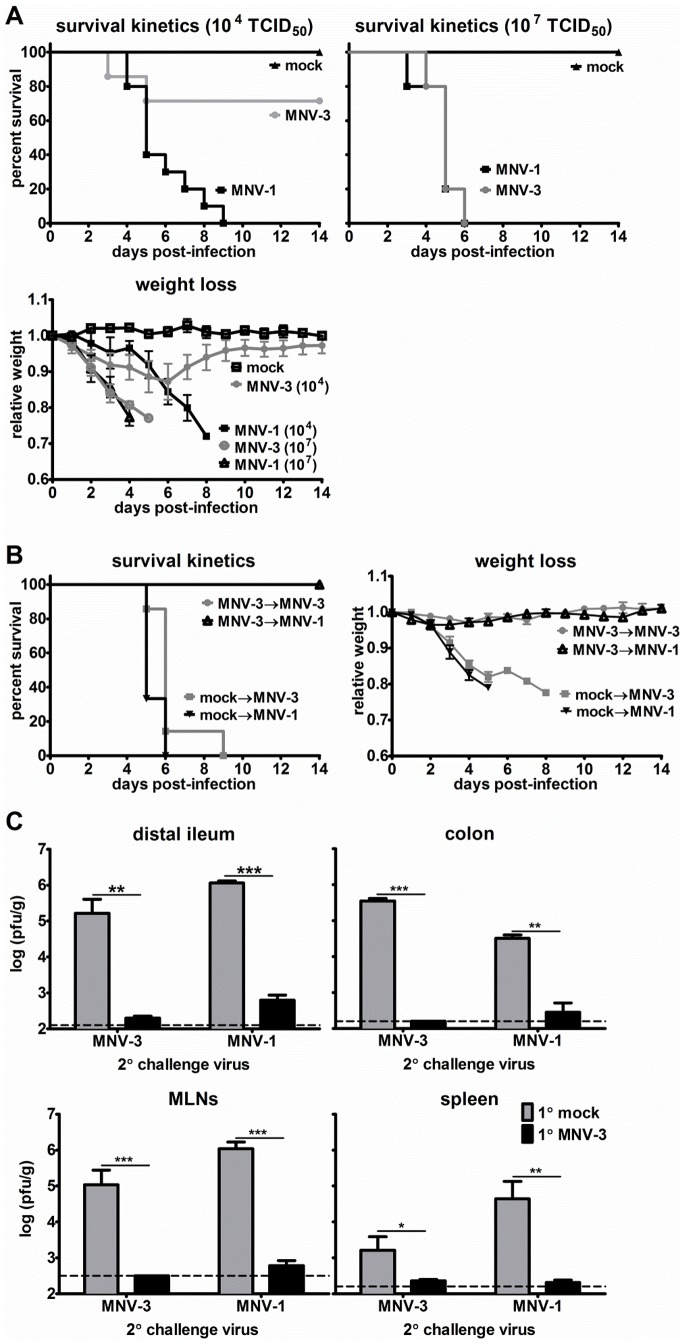
MNV-3 elicits protection from severe disease in the absence of type I interferon signaling. **A**) Groups of mice lacking the type I interferon receptor (IFNAR^−/−^; a minimum of 5 mice per condition) were inoculated with mock inoculum, 10^4^ or 10^7^ TCID_50_ units of MNV-1 or MNV-3 by the peroral route. The percentage of mice surviving infection was calculated daily. All surviving mice were weighed daily and the weights compared to day 0 weights to calculate a relative weight. **B**) Groups of IFNAR^−/−^ mice (n = 3–5) were inoculated with either mock inoculum or 5×10^3^ TCID_50_ units of MNV-3. Six weeks later, mice were challenged with 10^7^ TCID_50_ units of MNV-3 or 10^6^ TCID_50_ units of MNV-1 and monitored for survival and weight loss, as described above. **C**) Groups of IFNAR^−/−^ mice (n = 3) were inoculated with mock inoculum (1° mock; grey bars) or 5×10^3^ TCID_50_ units of MNV-3 (1° MNV-3; black bars) by the peroral route. Six weeks later, mice were infected with 10^7^ TCID_50_ units of MNV-1 or MNV-3 (2° challenge virus displayed on the x-axis). One day following 2° challenge, animals were perfused, the indicated organs harvested, and viral burden determined by plaque assay. Limits of detection are indicated by dashed lines. MLNs = mesenteric lymph nodes. Groups of mice receiving mock versus MNV-3-1° infection and the same 2° challenge virus were compared for statistical purposes.

Because we could not examine whether primary MNV-3 infection protects from the development of disease in wild-type mice, we next performed challenge studies in mice lacking a functional type I interferon (IFN) receptor, IFNAR^−/−^ mice, which are known to be susceptible to MNV-1-induced lethality [39]. We previously reported that MNV-3 is attenuated compared to MNV-1 in STAT1^−/−^ mice which also have defective type I IFN signaling, causing self-limited gastroenteritis from which all animals quickly recover [35]. This is supported by results in IFNAR^−/−^ mice presented in **Figure 2A** – while all mice infected with 10^4^ TCID_50_ units of MNV-1 succumbed to infection by 9 days post-infection (dpi), only 2 of 7 MNV-3-infected mice succumbed at this dose. However, at the higher dose of 10^7^ TCID_50_ units, MNV-1 and MNV-3 caused equivalently lethal disease. This dose dependency is also reflected in weight loss, with a majority of mice infected with 10^4^ TCID_50_ units of MNV-3 losing weight initially but recovering by 8 dpi; in contrast, mice infected with 10^4^ TCID_50_ units of MNV-1 or 10^7^ TCID_50_ units of either virus strain continued to lose weight until death. Having identified sublethal and lethal doses of MNV-3, we next performed a challenge study by inoculating mice with mock inoculum or 5×10^3^ TCID_50_ units of MNV-3 (a dose not expected to cause severe disease) and then challenging both groups with 10^7^ TCID_50_ units of MNV-3 six weeks later. As expected, all mock-inoculated, MNV-3-challenged animals lost significant weight and eventually succumbed to infection; in contrast, 100% of MNV-3-infected, MNV-3-challenged animals remained healthy and survived challenge (**Figure 2B**). Primary low-dose MNV-3 infection also provided full protection to a secondary high-dose MNV-1 infection as measured by survival kinetics and weight loss. Protection from weight loss and lethality correlated with significant reductions in virus titers in the distal ileum, colon, MLNs and spleen of re-challenged mice compared to mice experiencing primary infection (**Figure 2C**). We conclude that primary MNV-3 infection affords homotypic and heterotypic protection from disease. Moreover, these data also demonstrate that type I IFN is not required for MNV-3 protective immunity.

### B cells and CD4^+^ T cells are essential to MNV-3 protective immunity

**Figure 3:**
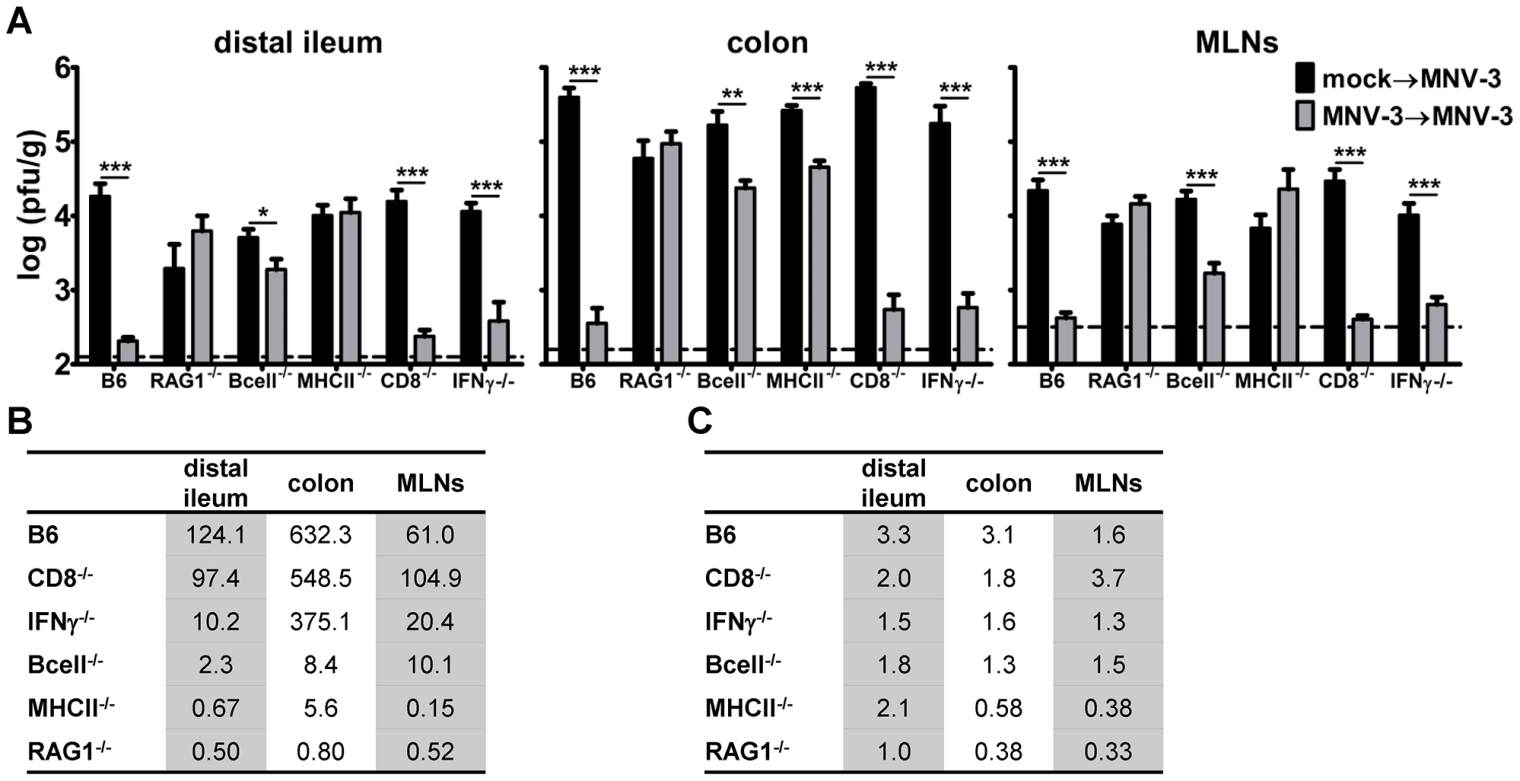
B cells and CD4^+^ T cells, but not CD8^+^ T cells and IFN-γ, are essential for MNV-3 protective immunity. **A**) Groups of mice of the indicated knockout strains (n = 7 mice per condition tested in at least two independent experiments) were inoculated with mock inoculum (black bars) or 10^4^ TCID_50_ units of MNV-3 (grey bars). Six weeks later, all mice were challenged with 10^7^ TCID_50_ units of MNV-3. One day later, animals were perfused, the indicated organs harvested, and viral burden determined by plaque assay. The data for all mice in each group were averaged. Limits of detection are indicated by dashed lines. The mock→MNV-3 infection group was compared to the MNV-3→ infection group for each mouse strain for statistical analysis. **B**) The mock→MNV-3 (1°) infection viral titers were divided by the MNV-3→ (2°) infection titers for the mice presented in panel A to determine the fold-reduction in titers as a quantitative measure of protective immunity. **C**) The same experiment was carried out for MNV-1; shown only are the fold-reductions in viral titers.

To gain insight into the memory immune mechanisms controlling MNV-3 re-challenges, we next performed homotypic MNV-3 challenge studies in a panel of knockout strains lacking specific components of the immune system (**Figures 3A and 3B**). As expected, we observed a complete loss of protection in RAG1^−/−^ mice. In fact, secondary titers were slightly higher in all tissues of RAG1^−/−^ mice compared to primary titers; this is not surprising since RAG1^−/−^ mice are known to be persistently infected with MNV-1 [40]. There were also significant reductions in MNV-3 protection in all tissues of mice lacking B cells and MHC class II molecules. On the other hand, mice lacking IFN-γ displayed only a modest reduction in MNV-3 protection and CD8^−/−^ mice were fully protected. These data unequivocally demonstrate that B cells and CD4^+^ T cells are essential to MNV-3 protection whereas CD8^+^ T cells are dispensable and IFN-γ plays only a modest role, at least under the infection conditions used in these experiments. Unexpectedly, we also observed significant differences when comparing primary infection titers of B6 mice and certain immunodeficient strains. For example, primary titers in all tissues of RAG1^−/−^ mice were significantly reduced compared to B6 mice; B cell^−/−^ mice had reduced distal ileum primary titers while MHC class II^−/−^ mice had reduced MLN primary titers. While we do not currently have an explanation for these observations, it is possible that the absence of B and/or T cells in these mice alters the numbers of resident mucosal macrophages and dendritic cells expected to be targets of MNV infection. The same homotypic challenge studies were performed in parallel for MNV-1 but based on the minimal protection observed in wild-type B6 mice, it was not possible to definitively measure a reduction in the magnitude of protection in knockout mouse strains (**Figure 3C**). These data do further highlight the remarkable difference in protective immunity elicited by MNV-1 and MNV-3.

### MNV-3 induces a more robust systemic and mucosal antibody response than MNV-1

**Figure 4:**
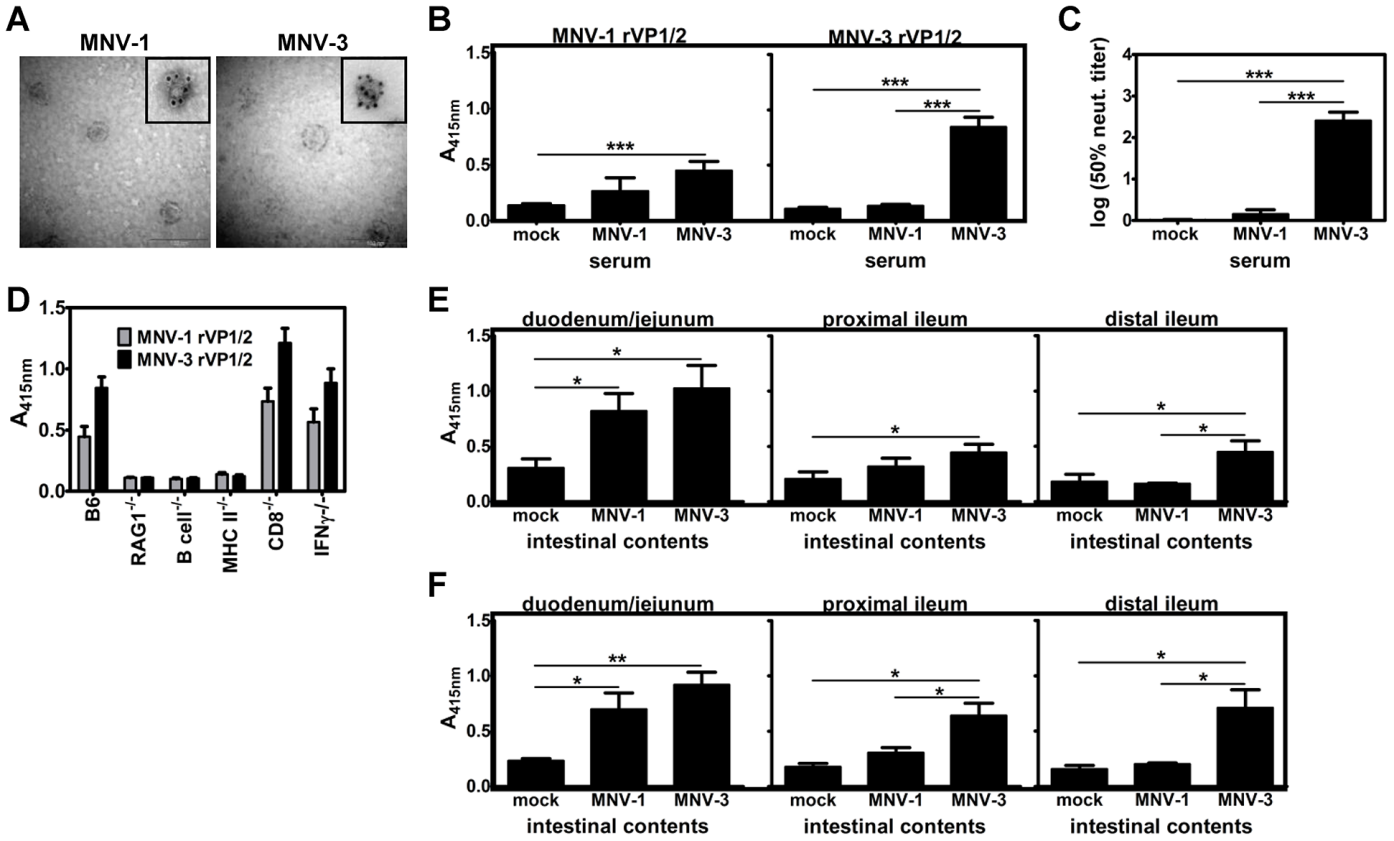
MNV-3 induces a more robust systemic and mucosal antibody response than MNV-1. **A**) Clarified supernatants of Sf9 cells infected with recombinant baculoviruses expressing MNV-1 or MNV-3 rVP1/2 were visualized by electron microscopy. The insets show representative VLPs stained with a polyclonal antibody to the MNV-1 VP1 protein using immunogold labeling. **B–F**) In panels B, C, E and F, groups of mice were inoculated with mock inoculum, 10^4^ TCID_50_ units of MNV-1, or 10^4^ TCID_50_ units of MNV-3. In panel D, only mice infected with MNV-3 were tested. In all cases, mice were challenged with homologous inoculum six weeks later. One day later, serum and intestinal contents were collected as described in the Methods. **B**) Serum samples from wild-type B6 mice (a minimum of 4 mice per condition tested in at least two independent experiments) were tested in ELISA on plates coated with the indicated rVP1/2. **C**) Serum was collected from groups of B6 mice (n = 3) inoculated with either mock inoculum or 10^4^ TCID_50_ units of the indicated MNV strain for six weeks. The 50% neutralization titer was determined for each serum sample. The entire experiment was repeated three times. Data from all samples per condition were averaged and compared for statistical purposes. **D**) Serum samples from groups of mice of the indicated knockout strain (a minimum of 4 mice per strain tested in at least two independent experiments) were tested in ELISA on plates coated with either MNV-1 (grey) or MNV-3 (black) rVP1/2. Intestinal contents from the indicated small intestinal piece of wild-type B6 mice (a minimum of 6 mice per condition tested in at least two independent experiments) were tested in ELISA on plates coated with MNV-1 rVP1/2 (**E**) or MNV-3 rVP1/2 (**F**). For all panels, the data for all mice in each group were averaged. Statistical analyses were carried out on the indicated groups as described in the Methods.

Based on the essential nature of B cells in mediating MNV-3 protection, we next tested whether MNV-3 induces a more robust antibody response than MNV-1. First, we collected serum from mice inoculated with mock inoculum or 10^4^ TCID_50_ units of MNV-1 or MNV-3 for six weeks and challenged with10^7^ TCID_50_ units of homologous virus for one day. These serum samples were tested against both MNV-1 and MNV-3 rVP1/2 as antigen in ELISA. This antigen was produced by expressing ORF2 and ORF3 of each virus strain in a baculovirus system, as described in the Methods. Representative electron microscopy images of MNV-1 and MNV-3 virus-like particles (VLPs) produced in infected Sf9 cells are shown in **Figure 4A**; the insets demonstrate immunogold labeling of representative VLPs with a polyclonal anti-MNV-1 VP1 antibody. While we could detect VLPs for both virus strains, they were not abundant and it is reasonable to assume that much of the VP1 and VP2 protein produced in this system was not in particle form; thus we feel it is more accurate to refer to the antigens as rVP1/2. MNV-3 induced a serum IgG response that reacted with homotypic rVP1/2 robustly and heterotypic MNV-1 rVP1/2 modestly; contrary to this, MNV-1 induced only a modest homotypic serum IgG response (**Figure 4B**). Furthermore, only MNV-3-specific serum neutralized homologous virus (**Figure 4C**). To further confirm the critical nature of virus-specific antibody in MNV-3 protection, we tested serum samples collected from knockout mice used in MNV-3 challenge studies. There was a direct correlation between the presence of virus-specific serum IgG (**Figure 4D**; present in wild-type, CD8^−/−^, and IFNγ^−/−^ strains; absent in RAG1^−/−^, B cell^−/−^, and MHC class II^−/−^ strains) and protection from a secondary challenge (see Figure 3). The virus-specific serum antibody response was significantly higher in CD8^−/−^ mice compared to wild-type B6 mice, possibly reflecting a compensatory enhancement in B cell responses in the absence of a functional CD8^+^ T cell response. Because of the enteric nature of NoVs, we next examined whether MNV-1 and MNV-3 differentially induce a mucosal IgA response. Specifically, intestinal contents (IC) were collected from three regions of the small intestine – the duodenum/jejunum, proximal ileum, and distal ileum – and tested in the rVP1/2-based ELISA using an anti-IgA secondary antibody. In all three intestinal segments, MNV-3-specific mucosal IgA levels were higher than MNV-1-specific levels against both MNV-1 (**Figure 4E**) and MNV-3 (**Figure 4F**) rVP1/2; this difference was statistically significant in the proximal and distal ileum. In fact, MNV-1-specific mucosal IgA was only significantly higher than background detection levels in the duodenum/jejunum segment whereas MNV-3-specific mucosal IgA was detectable in all three segments. This was true using either MNV-1 or MNV-3 rVP1/2 as antigen, consistent with the ability of MNV-3 to induce heterotypic protective immune responses.

### MNV-3-specific antibody is sufficient to mediate partial protection

**Figure 5:**
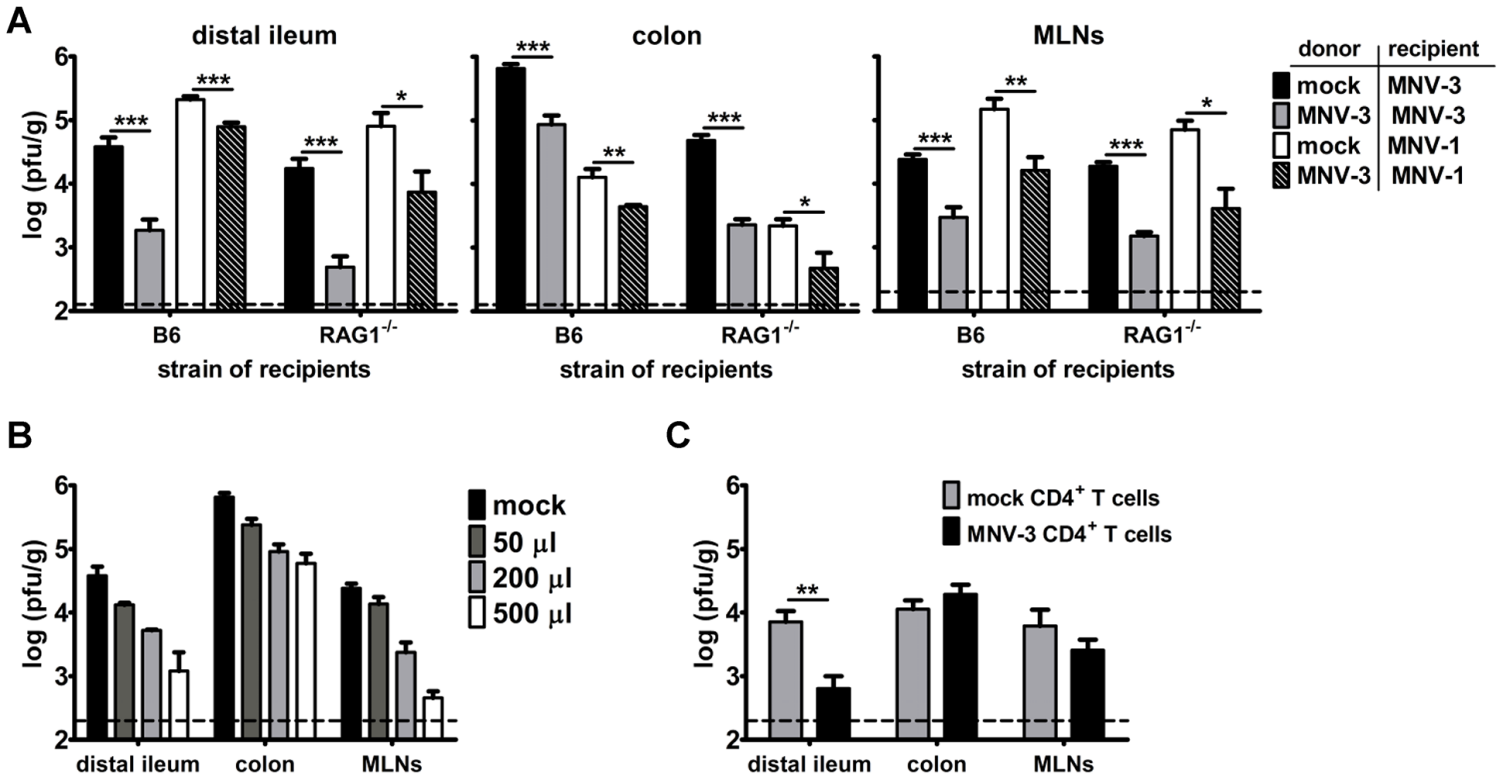
Antibody and CD4^+^ T cells from MNV-3-infected mice provide partial protection. **A**) Donor B6 mice were inoculated with mock inoculum or 10^4^ TCID_50_ units MNV-3. Six weeks later, serum was collected by cardiac puncture and 250 µl serum was injected intraperitoneally into groups of recipient B6 and RAG1^−/−^ mice (n = 6 per strain in two independent experiments). One day later, recipient mice were challenged with 10^7^ TCID_50_ units of MNV-3 or MNV-1. At 1 dpi, animals were perfused, the indicated organs harvested, and viral burden determined by plaque assay. Black bars indicate recipient mice that received mock serum and MNV-3 challenge; grey bars indicate recipient mice that received MNV-3-immune serum and homologous MNV-3 challenge; white bars indicate recipient mice that received mock serum and MNV-1 challenge; and black striped bars indicate recipient mice that received MNV-3-immune serum and heterologous MNV-1 challenge. The data for all mice in each group were averaged. Groups of recipient mice of the same mouse strain receiving mock versus MNV-3-immune serum and the same challenge virus were compared for statistical purposes. **B**) Groups of recipient B6 mice (n = 3) received 50, 200, or 500 µl MNV-3-immune serum and were challenged one day later with 10^7^ TCID_50_ units of MNV-3. Viral titers were determined in the indicated tissues at 1 dpi. The group of mice receiving mock serum and MNV-3 challenge from panel A is included here for clarity. **C**) 2×10^6^ CD4^+^ T cells collected from mock- or MNV-3-immunized donor B6 mice (grey and black bars, respectively) were injected into groups of recipient RAG1^−/−^ mice (n = 3) intraperitoneally. One day later, recipient mice were challenged with 10^7^ TCID_50_ units of MNV-3. At 1 dpi, the indicated tissues were harvested and viral loads were determined by plaque assay. The experiment was repeated two times and all data are averaged.

To test whether MNV-3-specific antibody is sufficient to mediate protection towards a primary MNV infection, we next carried out passive transfers of serum from mock-inoculated or MNV-3-infected mice. Specifically, 250 µl serum collected from mice that had been infected for six weeks was inoculated intraperitoneally into naïve B6 or RAG1^−/−^ recipients. At 1 d post-transfer, mice were infected with either MNV-1 or MNV-3 and assessed for virus loads one day later. In both B6 and RAG1^−/−^ recipient mice, passive transfer of serum from MNV-3-infected mice provided significant protection from MNV-3 and MNV-1 infections in the distal ileum, colon, and MLNs (**Figure 5A**). We performed MNV-1-specific serum transfers in parallel; as expected based on the very low level of virus-specific antibody detected by ELISA (see Figure 4B), no protection was afforded to either a homologous or a heterologous challenge (data not shown). Because 250 µl MNV-3-specific serum provided partial but not complete protection, we carried out a dosing study which clearly demonstrates a dose-dependent homotypic response (**Figure 5B**).

### MNV-3-specific CD4^+^ T cells are sufficient to mediate partial protection

We noted that MHC class II^−/−^ mice were completely incapable of mounting a protective immune response in the distal ileum or MLNs whereas B cell^−/−^ mice retained modest protection in these tissues; in contrast, both knockout strains retained modest protection in the colon (see Figure 3). Thus, we next tested whether CD4^+^ T cells play a helper-independent tissue-specific role in MNV protective immunity. To this end, we purified CD4^+^ T cells from the spleens of mock-inoculated or MNV-3-infected donor mice and adoptively transferred them into naïve RAG1^−/−^ recipients. The recipients were infected with MNV-3 1 d post-transfer and assessed for virus titers one day later. Interestingly, MNV-3-specific CD4^+^ T cells were capable of mediating significant protection in the distal ileum, although no protection was observed in the colons and only modest protection was observed in the MLNs (**Figure 5C**). Because MNV-3-specific CD4^+^ T cells provided small intestinal protection in RAG1^−/−^ recipients, we conclude that they are indeed capable of mediating helper-independent protection to MNV-3 infection in a tissue-specific manner.

### MNV-3 initiates replication faster than MNV-1 in vitro

**Figure 6:**
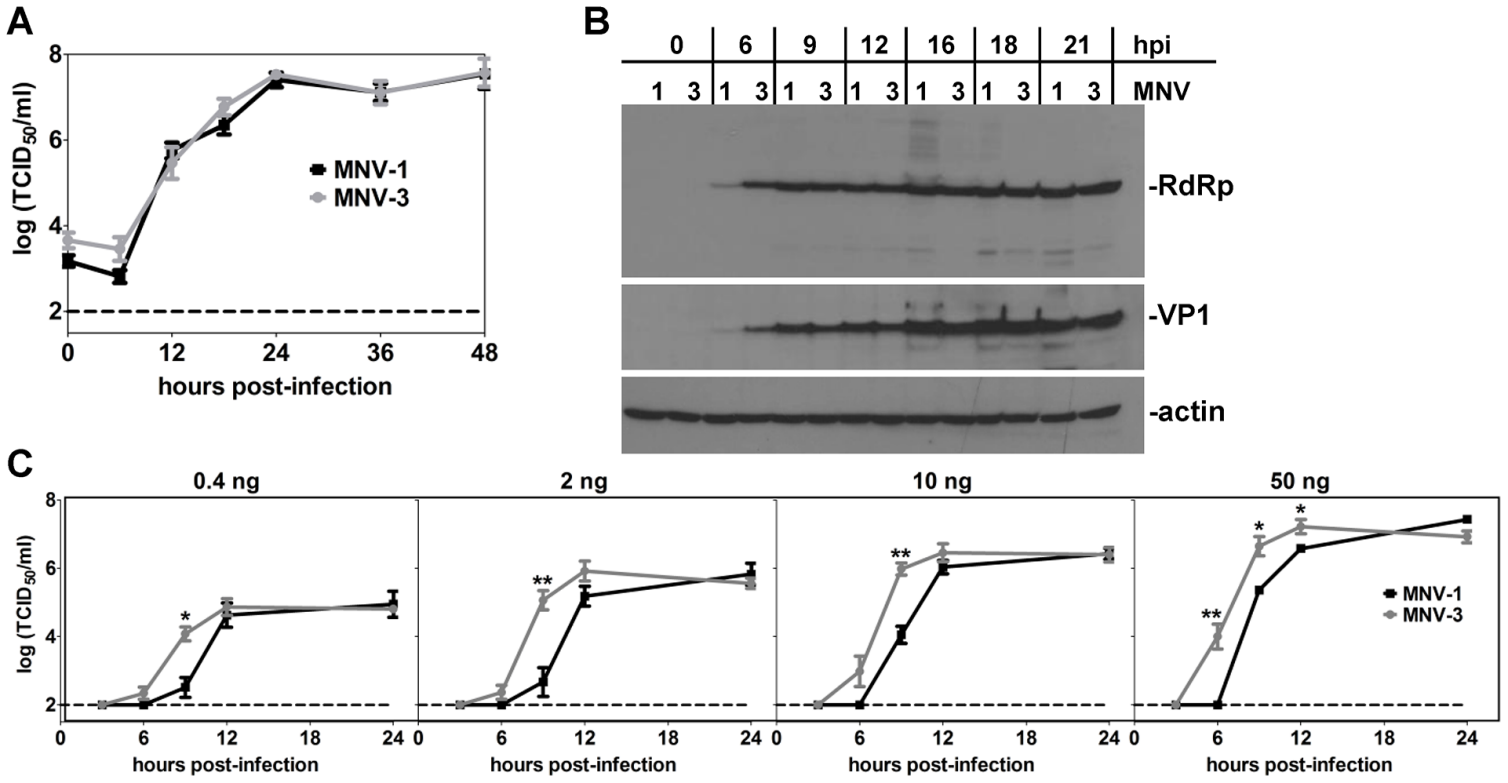
MNV-3 initiates replication faster than MNV-1. **A**) RAW 264.7 cells were infected with MNV-1 or MNV-3 at MOI 5. Supernatant was collected from two independent wells at the indicated hours post-infection (hpi) and virus titers determined using TCID_50_ assay. The entire experiment was repeated three times and data from all experiments are averaged. The limit of detection of the assay is indicated by a dashed line. **B**) Infected cells from the same cultures used for panel A were lysed and viral proteins were detected by western blotting using the indicated antibodies. These data are representative of duplicate samples tested from each of three independent experiments. **C**) 1.5×10^5^ HEK-293T cells were transfected with 0.4, 2, 10 or 50 ng of purified MNV-1 or MNV-3 genomic RNA. The virus titers at the indicated hpi were determined using TCID_50_ assay. Data for all replicates are averaged. Data for MNV-1 and MNV-3 at each time point were compared for statistical purposes.

To begin dissecting the mechanistic basis for the difference in MNV-1 and MNV-3 protective immunity, we performed a set of comparative molecular analyses of in vitro replication kinetics. Overall replication rates were similar between MNV-1 and MNV-3 in RAW 264.7 cells (**Figure 6A**) and they produced comparable levels of the viral RNA-dependent RNA polymerase (RdRp) and capsid/VP1 proteins over the course of infection (**Figure 6B**). We did note that MNV-3 RdRp and VP1 levels were elevated compared to MNV-1 levels at the earliest time of detection, namely 6 hpi. Although this increased protein production early in infection did not translate to faster virion production in infected cells, it did correlate with faster virion production in HEK-293T cells transfected with viral genomes (**Figure 6C**); HEK-293T cells are not permissive to MNV infection but support a single round of replication upon genome transfection. In fact, progeny MNV-3 could be detected in as little as 6 hpi in transfected cells whereas progeny MNV-1 was not detected until 9 hpi. The same pattern was observed in permissive RAW 264.7 cells transfected with viral genomes (data not shown).

### Chimeric viruses containing reciprocal ORF3 genes replicate normally

**Figure 7:**
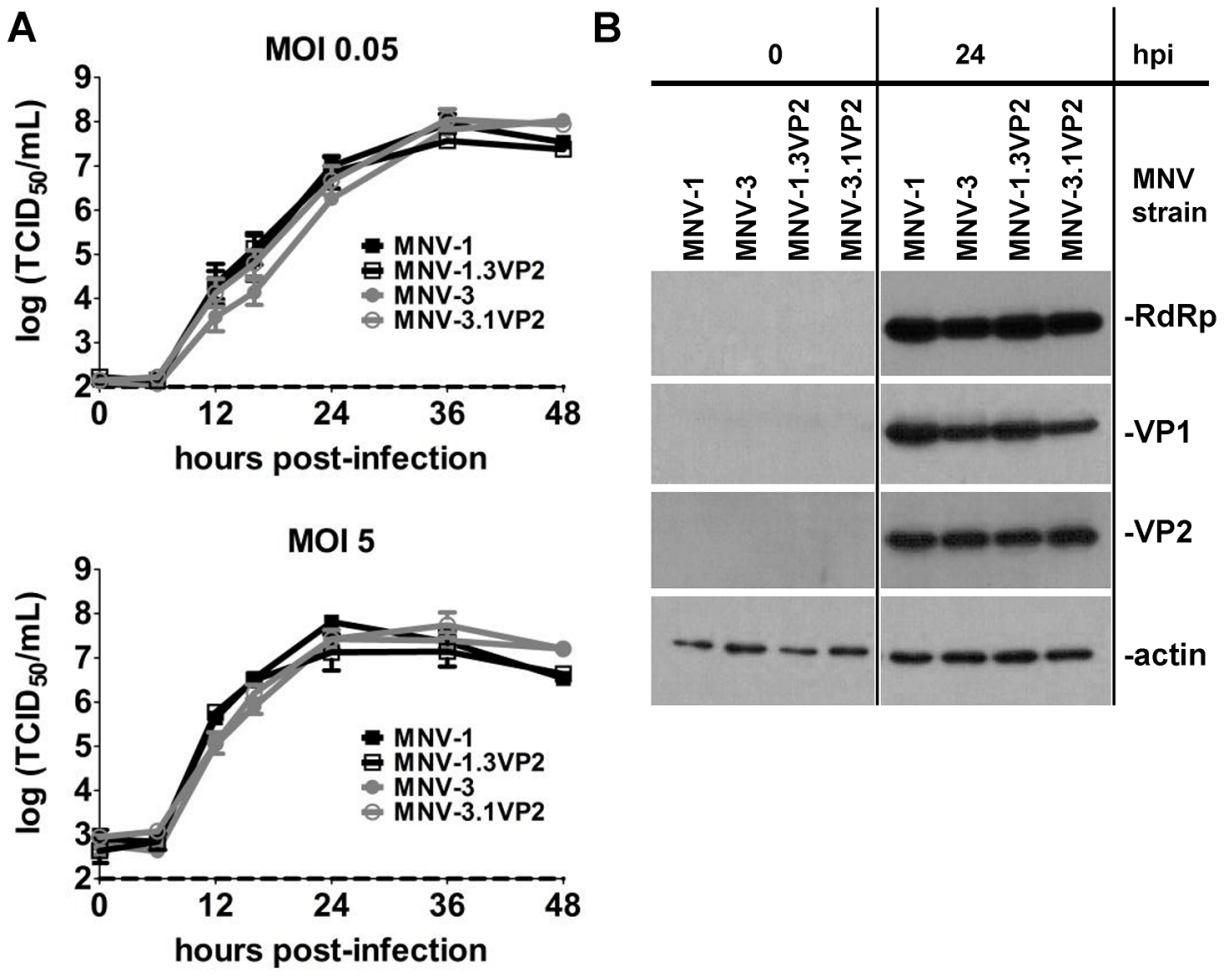
Recombinant ORF3-swap viruses replicate comparably to parental viruses. **A**) RAW 264.7 cells were infected with recombinant MNV-1, MNV-3, MNV-1.3VP2, or MNV3.1VP2 at MOI 0.05 or MOI 5 and growth curves were carried out as described in the legend of Figure 6A. Data from four independent experiments, with duplicate wells per condition tested in each experiment, are averaged. **B**) RAW 264.7 cells were infected with the indicated virus at MOI 5 for 0 or 24 hpi and cell lysates analyzed for viral proteins using western blotting. Data are representative of duplicate samples tested from each of three independent experiments.

In an effort to elucidate the viral determinants of protective immunity induction, we first examined the overall level of diversity across the viral genomes of MNV-1 and MNV-3. The ORF1 nonstructural proteins are 96.3% identical, the VP1 proteins are 94.6% identical, the VF1 proteins are 89.6% identical, and the VP2 minor structural proteins are 91.3% identical. Because a relatively high degree of variability was observed in the VP2 protein sequence, we generated chimeric viruses in which the ORF3 gene encoding VP2 was exchanged between parental MNV-1 and MNV-3; these were named MNV-1.3VP2 and MNV-3.1VP2. The parental and chimeric recombinant viruses replicated with similar kinetics in multi-step and single-step growth curves (**Figure 7A**), and produced generally comparable levels of RdRp and VP1 proteins (**Figure 7B**). We initially attempted to compare the levels of the VP2 protein itself with a polyclonal rabbit antibody raised to the MNV-1 VP2 whole protein [41] but this antibody did not appear to recognize the MNV-3 VP2 protein efficiently (data not shown). This was confirmed by transfecting cells with an expression plasmid encoding the MNV-1 or MNV-3 VP2 protein fused to a 6×-His marker. Although an anti-His antibody recognized both VP2.His fusion proteins, the anti-VP2 antibody recognized only the MNV-1 VP2.His protein (**Figure S1A**). Therefore, we generated a monospecific antibody to a peptide conserved between the MNV-1 and MNV-3 VP2 proteins (**Figure S1B**), which recognized MNV-1 and MNV-3 VP2 proteins equivalently (**Figure S1C**). To further rule out the possibility that MNV-1 and MNV-3 VP2 translation efficiency differed, we carried out an in vitro translation-termination (TTR) reporter assay since this is the mechanism by which VP2 translation is initiated [42]. Three nucleotide differences exist between MNV-1 and MNV-3 within the essential TTR region upstream of the VP2 start codon. We introduced one (1-U/C), two (1-UG/CA), or all three (WT-3) MNV-3 residues into a MNV-1 TTR reporter construct and verified that all three mutant constructs supported TTR comparable to the parental MNV-1 (WT-1) (**Figure S1D**). When using the newly generated monospecific anti-VP2 antibody, similar levels of VP2 protein were produced from each of our recombinant viruses (**Figure 7B**).

### MNV-1 and MNV-3 differentially induce cytokines independent of VP2

**Figure 8:**
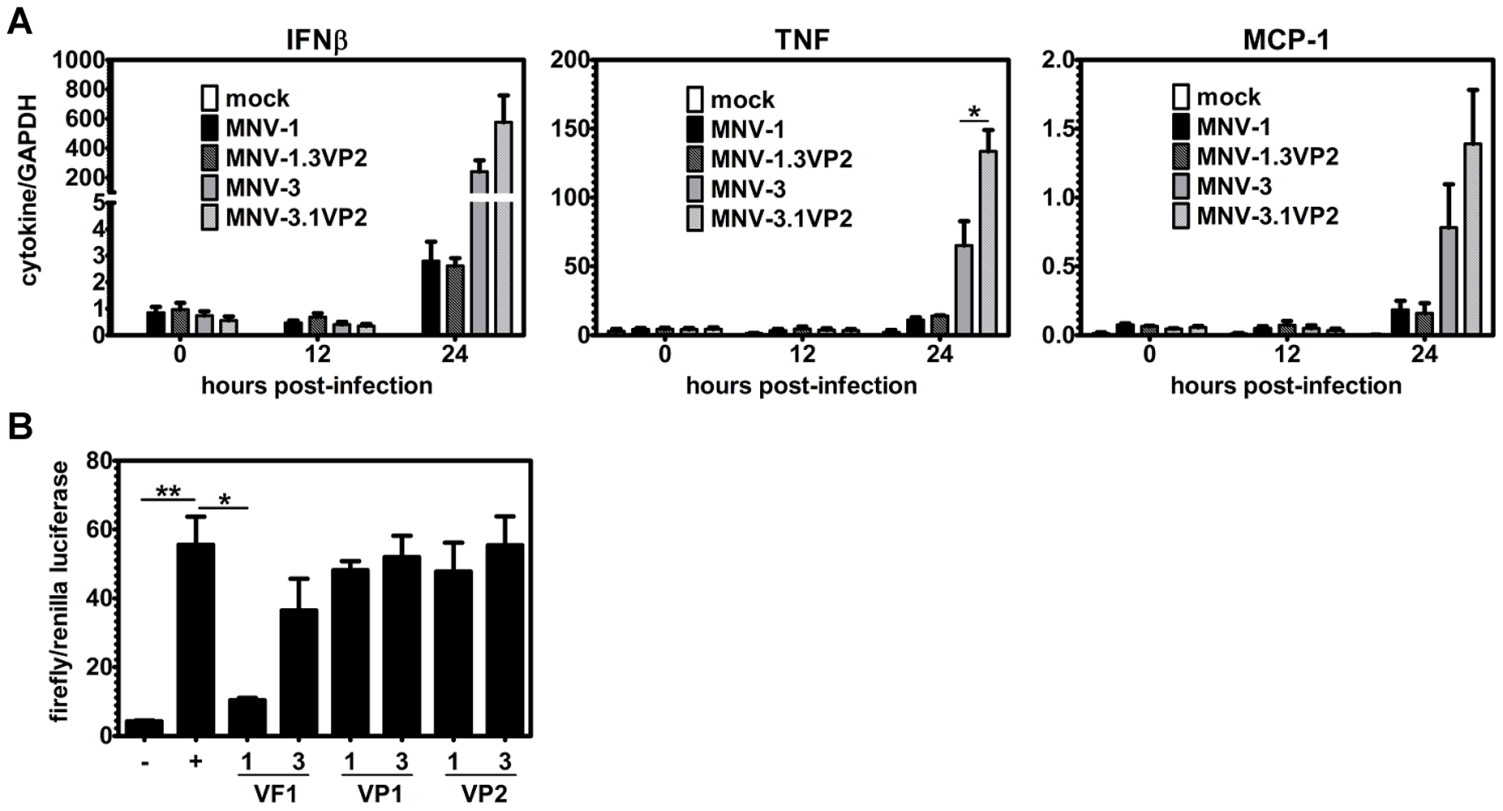
VF1 regulates cytokine induction in vitro. **A**) RAW 264.7 cells were infected with recombinant MNV-1, MNV-3, MNV-1.3VP2, or MNV3.1VP2 at MOI 5, or a mock inoculum. Total RNA was extracted from cells at 0, 12, and 24 hpi and cytokine expression determined through qRT-PCR, as described in the Methods. Data from duplicate wells per condition, and two independent experimental replicates, were averaged. **B**) HEK-293T cells were transfected with pβLUX, pRL-SV40, pN-RIGI and one of the following test plasmids – pTriEx-1ORF4 (VF1-1), pTriEx-3ORF4 (VF1-3), pTriEx-1ORF2 (VP1-1), pTriEx-3ORF2 (VP1-3), pTriEx-1ORF3 (VP2-1), or pTriEx-3ORF3 (VP2-3). In the negative control (−), only pβLUX, pRL-SV40, and filler DNA were transfected. In the positive control (+), the test plasmid was replaced with filler DNA. Each condition was tested in duplicate and the entire experiment repeated three times. Data represent the averaged readings from all experiments. Each group was compared to the positive control for statistical purposes.

To test the hypothesis that MNV-1 antagonizes cytokine expression, leading to impaired protective immunity induction – and if so, to test whether VP2 regulates this process – we infected RAW 264.7 cells with MNV-1, MNV-3, MNV-1.3VP2, or MNV-3.1VP2 and collected cell lysates at 0, 12, and 24 hpi. Supporting the hypothesis that cytokine antagonism is related to weak MNV-1 protective immunity induction, parental MNV-3 induced significantly higher levels of IFN-β, TNF, and MCP-1 transcripts than parental MNV-1 (**Figure 8A**). Related to the role of VP2 in this phenotype, MNV-3.1VP2 induced all cytokines to a similar magnitude as parental MNV-3 (and even induced higher levels of TNF), whereas MNV-1.3VP2 induced cytokines very weakly similar to parental MNV-1. We conclude that MNV-1 and MNV-3 unequivocally differ in their induction of cytokines in infected macrophage cultures, with protective MNV-3 inducing a significantly more robust cytokine and chemokine response than MNV-1. We also conclude that the VP2 protein does not regulate this process. Because MNV-1 VF1 has been shown to block IFN-β induction [5], we tested whether there are virus strain-specific differences in this activity. Using a luciferase reporter assay to measure IFN-β promoter activity (**Figure 8B**), we show that MNV-1 and MNV-3 VP1 and VP2 proteins lack IFN-β antagonist activity. We also confirm that MNV-1 VF1 possesses this activity, consistent with published data [5]. In contrast, MNV-3 VF1 did not block IFN-β promoter activation. Although we have yet to confirm that this difference is related to our protective immunity phenotype in vivo, it is reasonable to predict that the ability of NoV strains to antagonize cytokine induction will correlate with impaired protective immunity.

### VP2 regulates maturation of antigen presenting cells

**Figure 9:**
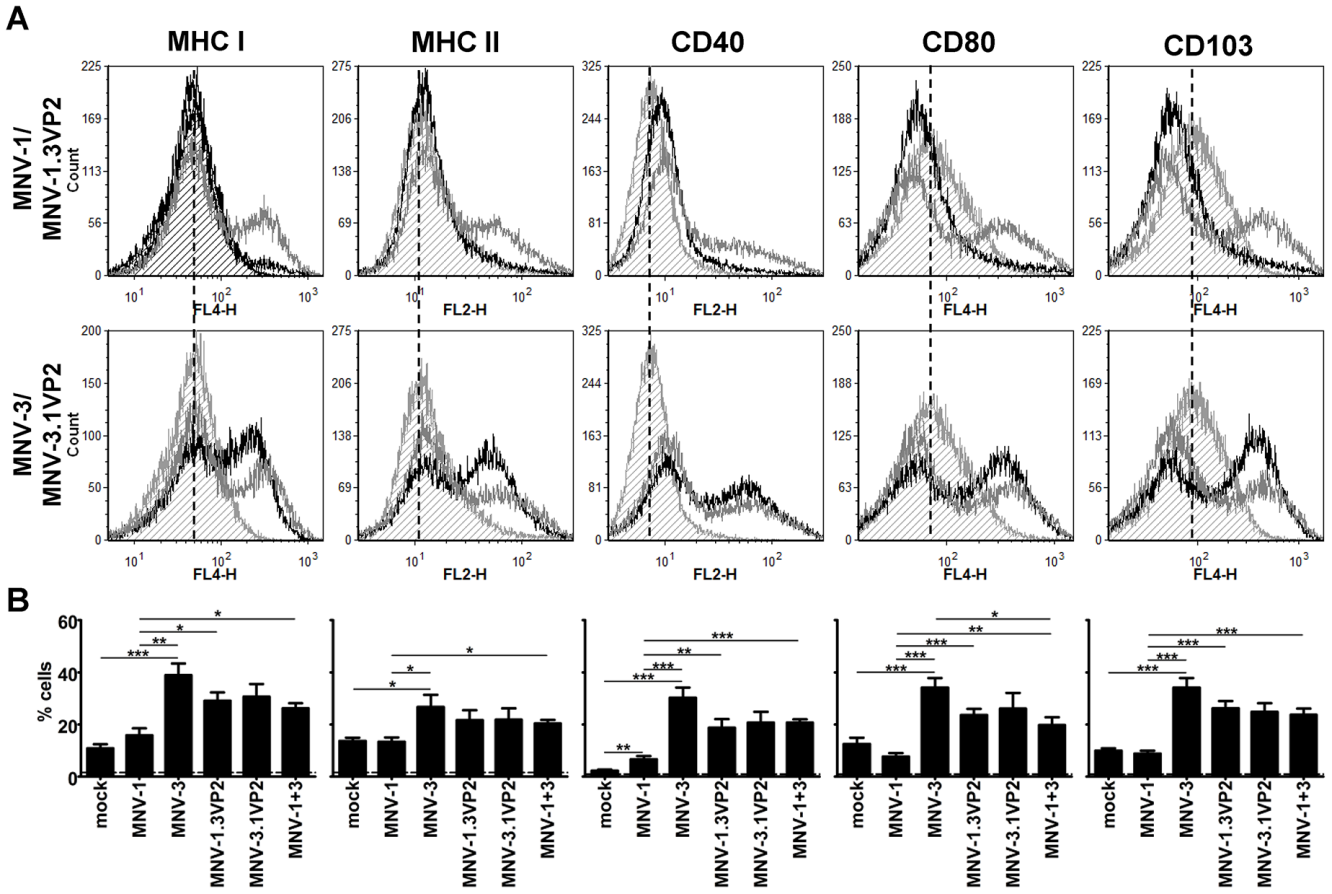
VP2 regulates antigen presenting cell maturation in vitro. 5.0×10^5^ RAW 264.7 cells were plated overnight in 24-well plates and then exposed to mock inoculum or recombinant MNV-1, MNV-3, MNV-1.3 VP2, or MNV-3.1 VP2 at MOI 5 for 24 h. In certain experiments, a well was also co-infected with equivalent titers of parental MNV-1 and MNV-3 such that cells were infected at MOI 5. Cells were stained with antibodies to MHC class I and MHC class II molecules, co-stimulatory molecules CD40 and CD80, or CD103. Flow cytometry was carried out as described in the Methods. Six independent experiments were performed for single infections, and three experiments included co-infections. **A**) Representative data from one experiment are presented in the histograms. In these graphs, surface expression of the indicated marker is shown for mock-inoculated cells (light grey histogram filled with dashed lines); cells infected with parental MNV-1 or MNV-3 (black histogram); and cells infected with chimeric MNV-1.3VP2 or MNV-3.1VP2 (dark grey histogram). The parental and chimeric virus pair is shown to the left of the histograms. The dashed vertical lines indicate the mean basal expression levels in mock-inoculated cells. **B**) In the bar graphs, the percentage of cells displaying upregulated surface expression of the indicated marker is presented. Data from all experimental replicates are averaged. Cells inoculated with UV-inactivated MNV-1 or MNV-3 displayed statistically similar levels of each marker compared to mock-inoculated cells (data not shown). Statistical comparisons were made between mock and each parental MNV; MNV-1 and MNV-3; each parental and chimeric pair; and each parental MNV and the co-infected cells.

Because MNVs are known to infect APCs including macrophages and dendritic cells [43], we were curious whether MNV-1 and MNV-3 also differed in their effect on APC maturation that is required for stimulation of T cell responses in vivo. To test this possibility, we infected RAW 264.7 cells with MNV-1 or MNV-3 and analyzed cell surface levels of antigen presentation molecules MHC class I and MHC class II, and co-stimulatory molecules CD40 and CD80. We also examined levels of surface CD103 – CD103 is expressed by mucosal APCs that imprint gut homing molecules on T and B cells in MLNs [44]. At 16 hpi, no increase in surface expression of these molecules was evident in either MNV-1- or MNV-3-infected cultures although the known stimulant poly(I:C) caused their robust up-regulation (data not shown). At 24 hpi however, a population of cells in MNV-3-infected cultures displayed increased MHC class I, MHC class II, CD40, CD80, and CD103 on their surfaces (**Figures 9A and 9B**). Viral replication was required for this process since UV-inactivated MNV-3 did not cause up-regulation (data not shown). In striking contrast to MNV-3 results, there was negligible up-regulation of any maturation marker in MNV-1-infected cultures at 24 hpi, revealing that MNV-1 avoids stimulation of APC maturation whereas MNV-3 does not. We also noted that both MNV-1 and MNV-3 caused a down-regulation of CD80 and CD103 on a population of cells (**Figure 9A**). To test whether VP2 regulates APC maturation, we also examined levels of maturation markers on cells infected with MNV-1.3VP2 or MNV-3.1VP2. MNV-1.3VP2-infected cultures contained a population of cells with increased cell surface expression of maturation markers compared to parental MNV-1, although this population was smaller than in MNV-3-infected cultures. MNV-3.1VP2-infected cultures contained fewer cells displaying up-regulated cell surface expression of the various markers than parental MNV-3-infected cultures, although this difference did not reach statistical significance. To test whether MNV-1 blocked MNV-3-induced APC maturation, we also co-infected cells with equivalent doses of each parental virus and measured levels of the maturation markers (**Figure 9B**). For all markers, surface expression levels in co-infected cells were significantly higher than in singly MNV-1-infected cells. Conversely, for all markers other than CD80, surface expression levels in co-infected cells were statistically similar to singly MNV-3-infected cells. Collectively, these data demonstrate that (i) protective MNV-3 causes APC maturation on a proportion of cells in infected cultures whereas non-protective MNV-1 avoids APC maturation; (ii) VP2 regulates APC maturation; and (iii) MNV-1 does not actively prevent MNV-3-induced APC maturation.

### VP2 contributes to MNV protective immunity

**Figure 10:**
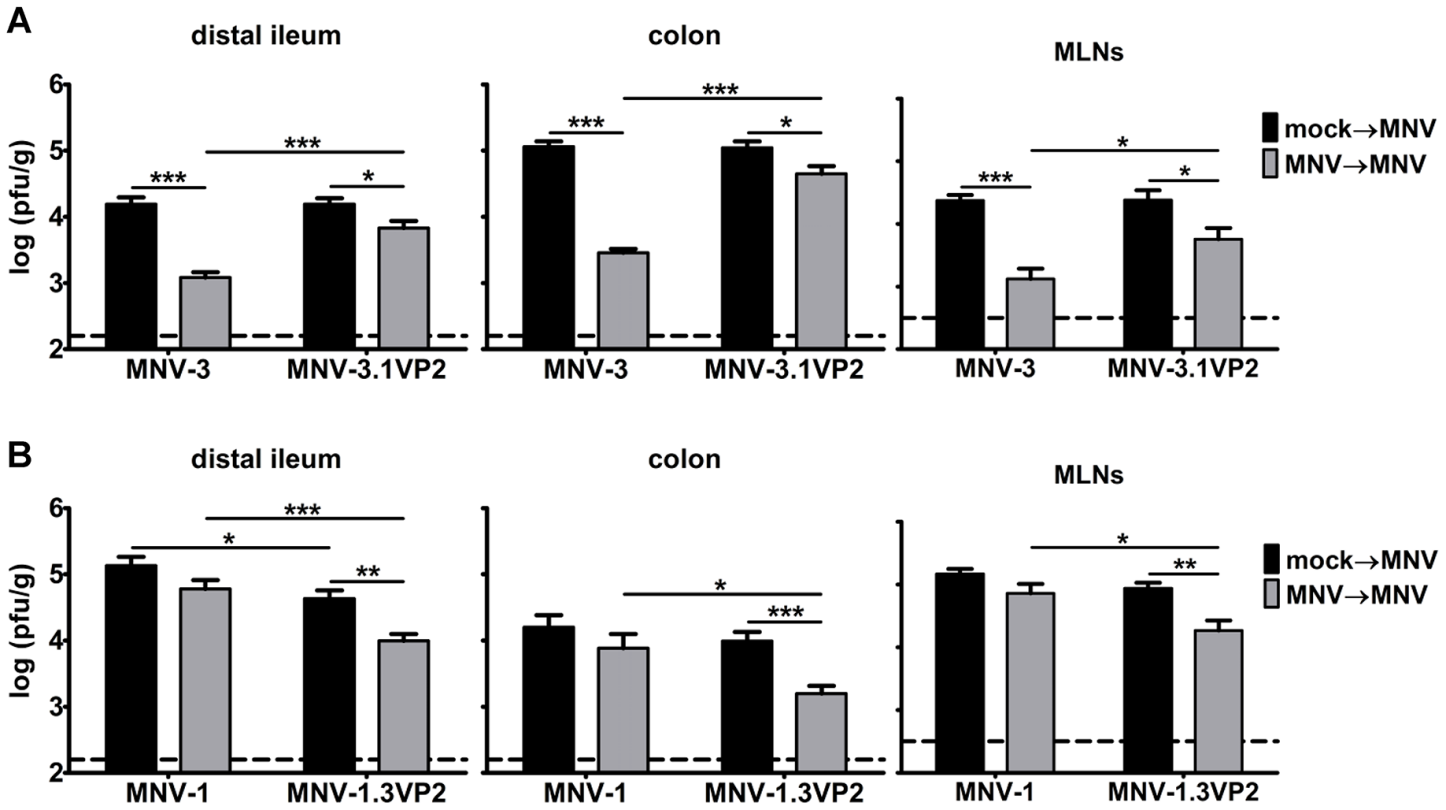
VP2 regulates MNV protective immunity. **A**) Groups of wild-type B6 mice (6 mice per group tested in two independent experiments) were inoculated with mock inoculum (black bars), or 10^4^ TCID_50_ units of recombinant MNV-3 or MNV-3.1VP2 (grey bars). Six weeks later, animals were challenged with 10^7^ TCID50 units of the same virus as indicated on the x-axis. One day later, mice were perfused, tissue samples were harvested, and viral loads were determined by plaque assay. **B**) The same experiment was carried out with recombinant MNV-1 and MNV-1.3VP2.

Based on our in vitro finding that VP2 regulates maturation of APCs, we next tested whether it contributes to protective immunity induction. We first performed protection studies using parental MNV-3 or MNV-3.1VP2 to determine whether the MNV-1 VP2 protein reduces the magnitude of MNV-3 protection. While all primary infection titers were comparable between the two viruses (**Figure 10A** **, black bars**), secondary infection titers were significantly higher for MNV-3.1VP2 in all three tissues (**Figure 10A** **, grey bars**; 6-fold higher in the distal ileum; 18-fold higher in the colon; and 4-fold higher in the MLNs), demonstrating reduced protective immunity. Furthermore, we observed significantly reduced secondary titers in all three tissues of mice receiving MNV-1.3VP2 compared to parental MNV-1 (7-fold lower in the distal ileum; 8-fold lower in the colon; and 3-fold lower in the MLNs), indicating enhanced protective immunity in the presence of MNV-3 VP2 (**Figure 10B**). Overall, we conclude that the minor structural protein VP2 is a major determinant of NoV protective immunity induction.

## Discussion

In this study we report the following four important observations related to NoV protective immunity: 1) NoV strains differing by as little as 5% in their capsid protein sequences – intra-cluster strains – can induce remarkably divergent levels of protective immunity; 2) the critical immune mediators of NoV protective immunity are antibody and CD4^+^ T cells, with CD4^+^ T cells playing a helper-independent role; 3) the viral minor structural protein VP2 is a determinant of NoV protective immunity that regulates APC maturation; and 4) antagonism of cytokine induction is likely another mechanism used by NoVs to avoid stimulating protective immunity.

### Intra-cluster NoV strains induce remarkably divergent levels of protective immunity

Norovirus protective immunity has become a controversial topic in recent years based on seemingly contradictory results gathered from human challenge studies and pandemic GII.4 epidemiological patterns. In human challenge studies carried out in 1973 [22] and 1990 [21], volunteers were inoculated with the prototype Norwalk virus (a GI.1 HuNoV) and then re-challenged with identical inoculum at various intervals later. The results of these studies demonstrated that at least a proportion of individuals failed to mount a lasting protective response to primary Norwalk infection. More recently, the epochal pattern of evolution of pandemic GII.4 HuNoVs strongly suggests the development of herd immunity in the population [23]–[25]. There are several possible explanations for this apparent discrepancy which are not mutually exclusive: First, HuNoVs may commonly induce short-term immunity capable of driving the evolution of new dominant strains, but this immunity is not long-lived. There is evidence for this from the human challenge studies in which protection was observed to last for only six months [21]. Moreover, we have demonstrated in the murine model of NoV infection that protection wanes substantially in mucosal tissues between six weeks and six months post-primary exposure (Figure 1B, and [7]). Second, there may be HuNoV strain-specific distinctions in protective immunity induction since early volunteer studies were all carried out with a GI.1 strain while recent pandemics have all been caused by GII.4 strains. In fact, our results presented here demonstrate that NoV strains differing by as little as 5% in their VP1 protein sequences can elicit remarkably disparate levels of protective immunity (Figure 1). Importantly, this is the same degree of VP1 similarity present amongst the pandemic GII.4 HuNoV strains and demonstrates unequivocally that even strains within the same NoV cluster can differentially interact with the host immune system.

Consistent with our previous results demonstrating that primary MNV-1 infection fails to protect mice from developing fecal inconsistency upon a secondary challenge [37], MNV-1 elicited weak to no protective immunity as defined by reduced secondary versus primary infection titers (Figure 3C; 3.3-fold, 3.1-fold, and 1.6-fold reductions in the distal ileum, colon, and MLNs, respectively, of B6 mice). In striking contrast, the relatively attenuated MNV-3 elicited robust protection in all tissues analyzed (Figure 3B; 124-fold, 632-fold, and 61-fold reductions in the distal ileum, colon, and MLNs, respectively). Primary MNV-3 infection not only elicited protection from a homotypic secondary challenge but also a heterotypic MNV-1 challenge (Figure 1). It is important to note that this difference in protection is not explained by a difference in the ability of these viruses to replicate in vivo. We have previously reported that MNV-1 and MNV-3 display a comparable time course of infection in wild-type mice, although MNV-3 actually reaches higher peak intestinal titers compared to MNV-1 [35]. Moreover, at the challenge dose of 10^7^ TCID_50_ units used in the current studies, MNV-1 and MNV-3 reached comparable primary infection titers in all tissues analyzed. One possible explanation for the failure of MNV-1 to induce protection is that it stimulates a locally suppressive or tolerogenic environment within the intestinal compartment. However, co-infecting with MNV-1 and MNV-3 did not prevent MNV-3 stimulation of homotypic or heterotypic protection (Figure 1), arguing against this possibility. Therefore, it is likely that MNV-1 either avoids detection by the host immune system or that it antagonizes the development of virus-specific adaptive immunity by directly affecting the infected APC. Our in vitro results support the latter possibility (see below). Interestingly, MNV-1 did not block the ability of MNV-3 to cause APC maturation in vitro, demonstrating that the activating phenotype of MNV-3 is dominant to the inhibitory phenotype of MNV-1 in this context.

Because the clearance rates of MNV strains have been reported to be variable [32], [34], [36], one consideration in our studies is that persistence affects the development of memory immune responses. In fact, MNV-3 has been reported to establish a prolonged infection compared to MNV-1 in B6 and CD-1 mice [32], [36]. It is therefore possible that sustained antigen exposure to the immune system during prolonged infections contributes to MNV protective immunity induction, although MNV-1 clearly encodes immune evasion/antagonism mechanisms as well. It should be noted that the MNV-1 and MNV-3 isolates used in our experiments are both cleared from 129SvEv mice by 7 dpi [35], in contrast to other published work. There are several possible explanations for the discrepancy between our results regarding MNV-3 clearance kinetics and other published work [32], [36] that we are actively investigating in independent studies in the lab. One possibility is that persistence is mouse strain-specific. Another possibility is that genetic differences between MNV-3 isolates used by different labs accounts for differential clearance rates. There are six amino acid differences between our MNV-3 isolate and that used by Arias et al. [36] that could account for this difference. Importantly, both MNV-3 isolates contain a glutamic acid at position 94 of NS1/2 which has been reported to be associated with persistent MNV infections [45], suggesting that additional factors may influence the ability of MNV strains to establish persistence. It will be interesting in future studies to elucidate the contribution of persistent MNV infection to the development of protective immunity.

### MNV-3 elicits protection from disease independent of type I IFN

Although we could not address whether MNV-3 elicits protection from disease in wild-type mice since MNV-3 is avirulent and MNV-1 induces only modest fecal inconsistency [35], [37], we can measure disease in IFN-deficient strains. Here we demonstrate that sublethal primary MNV-3 infection fully protected IFNAR^−/−^ mice from weight loss and lethality upon a high-dose secondary MNV-1 or MNV-3 challenge (Figure 2B). Moreover, very little infectious virus was detectable from tissues of re-challenged mice demonstrating robust memory immunity even in the absence of type I IFN (Figure 2C). These data unequivocally demonstrate that MNV-3 protective immunity is capable of preventing disease. They also indicate that type I IFN signaling is not required for MNV-3 protective immune induction, a result that is surprising based on the known role of type I IFN to enhance antigen presentation to T cells. It remains possible that type I IFN plays a more subtle role in MNV-3 induction of memory immune responses that is not reflected by weight loss, survival kinetics, and tissue titers at 1 d post-challenge.

### B cells and CD4^+^ T cells are critical for MNV protective immunity

To identify components of the host immune system required for MNV-3 protection, we used a panel of mouse strains genetically deficient in specific immune factors. These experiments revealed B cells and CD4^+^ T cells as absolutely essential to protection (Figure 3). Surprisingly, CD8^+^ T cells were completely dispensable for protection while IFN-γ played only a minor role. These results are not fully consistent with those of Chachu et al. where it was reported that B cells, CD4^+^ T cells, and CD8^+^ T cells all contribute to MNV-1 protection in a tissue-specific manner but none play an essential role [38]. Several experimental variables may contribute to the discrepancy between our results and those of Chachu et al., including virus strain (MNV-3 versus MNV-1, respectively) and number of previous exposures to the virus (one and two, respectively). Thus, virus strain-specific differences and/or repeated exposure to a NoV may alter the nature of the virus-specific memory immune response. Our studies were specifically designed to probe the host immune response to a primary NoV infection in order to explore potential explanations for seemingly contradictory observations regarding HuNoV protective immunity. It is not altogether unexpected that these responses would be qualitatively and quantitatively distinct from responses to a prime-boost model of vaccination. In light of the knowledge that people are recurrently exposed to natural NoV infections [6], [20], [30], it is critical to not only understand the immune response to vaccine regimens but also to natural infections which will likely shape the nature of a subsequent vaccine response. Thus, we believe these two sets of studies address distinct and equally fundamental issues in the NoV field.

### MNV-3 induces robust mucosal IgA and serum IgG, and virus-specific antibody confers protection in passive transfers

Based on the essential nature of B cells to MNV-3 protective immunity, we predicted that MNV-3 stimulates a stronger antibody response than MNV-1. Our ELISA and passive transfer data support this idea. First, MNV-3 induced significantly higher MNV-3 rVP1/2-reactive serum IgG, and even modestly higher MNV-1 rVP1/2-reactive serum IgG, than MNV-1 in wild-type mice (Figure 4B); only MNV-3-specific serum could neutralize homologous virus in vitro (Figure 4C). The minimal MNV-1-induced serum IgG was surprising in light of our and others' previous reports that MNV-1 induces a more robust serum IgG response [37], [40], [46]; the nature of the antigen and the inoculum dose are putative variables that could account for this difference. The low amount of MNV-1 rVP1/2-reactive IgG in the sera of MNV-3-infected mice was also somewhat surprising considering the robust heterotypic protection elicited by MNV-3 in challenge studies, suggesting that virus-specific serum IgG titers do not directly correlate with the magnitude of NoV protective immunity. Second, MNV-3 induced a generally more robust mucosal IgA response than MNV-1 (Figures 4E and 4F). While both virus strains induced detectable mucosal IgA in the duodenum/jejunum, MNV-3 levels were slightly, although not statistically, higher. Furthermore, only MNV-3-specific mucosal IgA was detectable in IC collected from the ileums of infected mice. This antibody reacted with both homotypic and heterotypic rVP1/2. This is, to our knowledge, the first report of detectable mucosal IgA to live MNV infection. Finally, passive transfer of serum from MNV-3-immunized mice mediated partial protection from both homotypic and heterotypic challenges even in RAG1^−/−^ recipients (Figures 5A and 5B).

### MNV-3-specific CD4^+^ T cells confer protection in a helper-independent manner

Because MHC class II-dependent responses were even more critical for MNV-3 protective immunity than B cell responses based on studies in knockout mice (Figure 3), we speculated that CD4^+^ T cells are required not only to provide help to B cells in our system but also in a helper-independent manner. Our adoptive transfer study supports this since purified CD4^+^ T cells were able to mediate partial protection to a MNV-3 primary infection even when recipient mice lacked other adaptive immune cells (i.e. RAG1^−/−^ recipients; Figure 5C). Interestingly, this helper-independent role of CD4^+^ T cells appears to be tissue-specific: While MHC class II^−/−^ mice completely lacked protection from MNV-3 re-challenge in the distal ileum and MLNs, they maintained modest protection in the colon comparable to the level seen in B cell^−/−^ mice. Moreover, adoptively transferred MNV-3-specific CD4^+^ T cells afforded partial protection in the distal ileum but not the colon. This tissue-specific phenotype is suggestive of a role for T_H_17 cells which migrate more readily to the small intestine than the colon [47], a possibility we are currently investigating.

### The viral minor structural protein VP2 regulates maturation of antigen presenting cells while VF1 regulates cytokine induction

Based on the high degree of divergence between the MNV-1 and MNV-3 VP2 protein sequences relative to other viral proteins, we tested the hypothesis that VP2 regulates protective immunity induction. The NoV VP2 protein is a small, highly basic protein that is present in 1–2 copies per virion, thus it is considered a minor structural protein [48], [49]. It has recently been shown to interact directly with the major capsid protein VP1 at a position supporting its localization in the interior of the capsid [4]. Based on this localization and its basic nature, it has been proposed to function in genome packaging. In vitro studies have also demonstrated that it aids in the expression and stability of VP1 and the viral particle [50], [51]. Finally, recent work demonstrates that VP2 negatively regulates the activity of the viral RdRp [52]. Our data suggest that VP2 plays an additional nonstructural role, regulating the host immune response to NoV infection. Specifically, we observed that VP2 regulates APC maturation (Figure 9): While MNV-3 caused up-regulation of MHC class I, MHC class II, CD40, CD80, and CD103 on a proportion of cells in infected cultures, MNV-1 failed to up-regulate any maturation marker. MNV-1.3VP2 displayed enhanced APC maturation compared to parental MNV-1 while MNV-3.1VP2 displayed reduced APC maturation compared to parental MNV-3. Interestingly, all four viruses caused a down-regulation of CD80 and CD103 on a population of cells. It was surprising to observe viral regulation of CD103 surface expression; to our knowledge, this is a novel observation. While CD103^+^ APCs have been most widely associated with tolerance induction, they may also stimulate protective immune responses at mucosal surfaces [44]. Determining the in vivo significance of viral regulation of CD103 may offer clues to the APC subsets involved in stimulating NoV protective immunity. In future studies, we plan to develop a method to distinguish between infected and bystander cells to test the notion that CD80 and CD103 down-regulation occurs specifically in infected cells. It will also be important to uncover the precise mechanism by which the MNV-1 VP2 protein antagonizes APC maturation.

In addition to differential APC maturation, we also observed a striking virus strain-specific difference in cytokine and chemokine induction (Figure 8). Specifically, protective MNV-3 induced IFN-β, TNF, and MCP-1 in infected macrophage cultures whereas non-protective MNV-1 induced only minimal amounts of each transcript. While VP2 does not regulate this process, it is interesting to speculate that the newly defined VF1 encoded by ORF4 is responsible. Similar to VP2, VF1 is relatively divergent in sequence between MNV-1 and MNV-3 compared to other viral proteins. More importantly, McFadden et al. recently reported that MNV-1 VF1 antagonizes expression of IFN-β in addition to several other antiviral genes [5]. We provide support for this possibility since the MNV-3 VF1 protein failed to block IFN-β promoter activation under conditions where the MNV-1 VF1 protein efficiently blocked activation (Figure 8B). Although HuNoVs do not encode a VF1, it is reasonable to predict that they encode distinct mechanisms to antagonize cytokine induction and that this antagonism is related to weak protective immunity of certain strains. Although our in vivo data suggest that type I IFN is dispensable for MNV-3 protective immunity (Figure 2), other cytokines that are differentially regulated by NoV strains could play a major role in regulating this process.

### VP2 is a determinant of norovirus protective immunity induction

Having determined that VP2 regulates APC maturation, a logical hypothesis is that it also regulates protective immunity induction in vivo. Indeed, we observed a reduction in protective immunity for MNV-3.1VP2 compared to parental MNV-3, and enhanced protective immunity for MNV-1.3VP2 compared to parental MNV-1 (Figure 10). Our data suggest that VP2 is a major, but not the sole, determinant of protective immunity induction since both chimeric viruses displayed an intermediate phenotype compared to parental MNV-1 and MNV-3. It is highly likely that cytokine induction also contributes to the generation of adaptive immune responses so future generation of ORF3/ORF4 chimeric viruses may facilitate a complete understanding of the mechanisms used by MNV-1 to avoid stimulating protective immunity.

### Conclusions

(1) Our data demonstrate a critical role for virus-specific antibody in mediating protection to a secondary NoV infection, and a clear virus strain-specific distinction in the antibody response to infection that is independent of in vivo levels of primary virus replication. Future efforts will focus on identifying virus epitopes targeted by neutralizing mucosal antibodies and elucidating why MNV-1 fails to elicit a more robust antibody response. (2) Our data also clearly define a helper-independent tissue-specific role for CD4^+^ T cells in NoV protective immunity. We are currently performing studies to elucidate the relevant CD4^+^ T cell subset, representing a novel target of vaccination. (3) In addition to these immune determinants of NoV protective immunity, we have revealed that the minor structural protein VP2 is a viral determinant of memory immune response stimulation. Mechanistically, the ability of VP2 to regulate APC maturation correlates with the magnitude of protection elicited by a MNV strain. (4) Finally, we have uncovered antagonism of cytokine induction as a correlate of impaired NoV protective immunity; this virus strategy is independent of VP2 and likely regulated by the newly described VF1. Overall, we conclude that NoVs have evolved at least two strategies to avoid stimulating adaptive immune responses – blocking APC maturation through a process involving VP2, and antagonizing cytokine induction. It is interesting to speculate that these immune evasion or antagonism strategies are also related to virulence since MNV-1 causes more severe disease than MNV-3 during a primary infection [35]. Ultimately understanding the mechanism by which certain NoV strains prevent the development of protective immunity is critical to effective design of a vaccine that requires frequent reformulations, as is expected of a HuNoV vaccine.

## Materials and Methods

### Cells and viruses

RAW 264.7 cells (ATCC) and HEK-293T cells (kindly provided by Dr. Fanxiu Zhu, Florida State University) were used for virus cultivation. MNV strains MNV-1.CW3 at passage 6 (referred to as MNV-1 herein) and MNV-3 at passage 6 (referred to as MNV-3) were used in all experiments [32], [43], either uncloned (Figures 1–6) or recombinant (Figures 7–10) versions. For the construction of recombinant viruses, total RNA was isolated from RAW 264.7 cells infected with MNV-1 or MNV-3 and cDNA was synthesized. Two overlapping fragments of the MNV-1 and MNV-3 genomes were amplified using the high fidelity PfuUltra II polymerase (Agilent Technologies). For cloning of the 5′ end, we used the sense primer 5′- GGAATTCCATATGTAGGCGTGTACGGTGGGAGGCCTATATAAGCAGAGCTCGTTTAGTGAACCGGT*GTGAAATGAGGATGGCAACGC*-3′ which contains a *Nde*I site, a minimal CMV promoter (underlined), an *Age*I site, and the conserved 5′ 21 nucleotides (nt) of the MNV genome (italics); and the antisense primer 5′-GGTACCTGAAATTGGCGTGTCTTG-3′ for MNV-1 (corresponding to nt 4234–4257 of the viral genome) or 5′-GCGAATCATGGTGCCAAGGTCAGA-3′ for MNV-3 (nt 4104–4127). The resulting PCR products were cloned into pCR-Blunt (Life Technologies) and confirmed by sequencing. These constructs were named pCRMNV-1.5 and pCRMNV-3.5. For cloning of the 3′ end, we used the antisense primer 5′-GCGTTAACGCGGCCGCTTTTTTTTTTTTTTTTTT-3′ which contains *Not*I and *Hpa*I sites (underlined) and an 18-nt thymidine repeat; and the sense primer 5′- GGAATTCCATATG *GCGGCAAGTACTCCATTGATGATTAC*-3′ which contains a *Nde*I site (underlined) and nt 2725–2750 of the viral genome (italics). The PCR products were gel-purified, digested with *Hpa*I and *Nde*I, and cloned into pSP73 (Promega). These constructs were named pSPMNV-1.3 and pSPMNV-3.3. To insert a hepatitis delta ribozyme sequence at the 3′ end of the genome [41], we amplified this cassette from pMNV* (kindly provided by Dr. Herbert Virgin, Washington University School of Medicine) using sense 5′-AAAAAAAAAAAAAAAAAAGGCCGGCATGGTCCCAGC-3′ and antisense 5′-GCGTTAACATGGAATAAGGAATGGACAGCAGG-3′ (ribozyme sequences underlined) primers. Fusion PCR was used to introduce the cassette into pSPMNV-1.3 and pSPMNV-3.3. For generating a full-length MNV-1 clone, pCRMNV-1.5 was digested with *Nde*I and *Hind*III (the MNV-1 genome has a *Hind*III site at nt 3710) and ligated to *Nde*I/*Hind*III-digested pSPMNV-1.3. For generating a full-length MNV-3 clone, pCRMNV-3.5 was digested with *Nde*I and *Eco*RI (the MNV-3 genome has an *Eco*RI site at nt 3123) and ligated to *Nde*I-*Eco*RI-digested pSPMNV-3.3. A synonymous G6390A nucleotide substitution was introduced into pSPMNV-3 using the QuikChange Lightning Site-Directed Mutagenesis Kit (Agilent Technologies) to delete a *Sac*II restriction site. The full-length MNV-1 and MNV-3 clones were designated pSPMNV-1.CW3 and pSPMNV-3, respectively. The GenBank accession numbers for these virus isolates are KC782764 (MNV-1) and KC792553 (MNV-3). Generation of chimeric MNV constructs has been described previously [53]. Briefly, MNV-1 and MNV-3 ORF3 genes were amplified from pSPMNV-1.CW3 and pSPMNV-3, gel-purified, and 1 µg of PCR product was used as the primer for mutagenesis reactions of the reciprocal parental template with the QuikChange Lightning Site-Directed Mutagenesis Kit. Chimeric clones, designated pSPMNV-1.3VP2 and pSPMNV-3.1VP2, were verified by sequencing.

### Virus stock generation

Uncloned virus stocks were generated from RAW 264.7 cell lysates using a sucrose cushion and cesium chloride gradient purification, as previously described [35], [43], [54], [55]. For generating stocks of recombinant viruses, HEK-293T cells were seeded at 10^6^ cells per well in a 6-well culture dish and cultured overnight. The cells were then transfected with 5 µg pSPMNV-1.CW3, pSPMNV-3, pSPMNV-1.3VP2, or pSPMNV-3.1VP2 using Lipofectamine 2000 (Life Technologies). At 24 h post-transfection, the cells were lysed by freeze-thaw at −80°C and supernatants clarified by centrifugation. The recombinant MNV-1, MNV-3, MNV-1.3VP2 and MNV-3.1VP2 viruses were passed in RAW 264.7 cells one time at MOI 0.05. Full-length genomic sequences were determined for all recombinant virus stocks and confirmed to contain no deviations from the original clones. All virus stocks were titrated by a standard TCID_50_ assay [34], [35]. A mock inoculum stock was prepared in parallel using RAW 264.7 cell lysate from uninfected cultures.

### Mice and infections

All mice were bred and housed in animal facilities at the University of Florida under specific-pathogen-free conditions. Wild type C57BL/6J (Jackson #000664; referred to as B6), B6RAG1^−/−^ (Jackson #002216; referred to as RAG1^−/−^), B cell^−/−^ (Jackson #002288), MHC class II^−/−^ (Jackson #003584; referred to as MHCII^−/−^), CD8^−/−^ (Jackson #002665), and IFNγ^−/−^ (Jackson #002287) mice were purchased from The Jackson Laboratory (Bar Harbor, ME). The Ifnar^1tm1Agt^ (referred to as IFNAR^−/−^) mice were provided by Dr. Herbert W. Virgin (Washington University School of Medicine) [39]. For all experiments, eight- to ten-week old, sex-matched mice were inoculated perorally with 25 µl inoculum. For virus load determination in specified tissues, mice were perfused and tissues dissected, weighed, and homogenized in media by bead beating using 1.0 mm zirconia/silica beads (BioSpec Products, Inc.). Plaque assays of tissue samples were performed as previously described [35], [43]. Cardiac puncture was used to collect blood which was separated by centrifugation in serum separator tubes. For collection of intestinal contents (IC), 1.5 inches of the small intestine immediately adjacent to the stomach (duodenum/jejunum), 1.5 inches of the proximal ileum, and 1.5 inches of the distal ileum were dissected and each segment was flushed with 600 µl of cold PBS supplemented with a protease inhibitor cocktail (Sigma, St. Louis, MO).

### Ethics statement

All animal experiments were performed in strict accordance with federal and university guidelines. Specifically, we adhered to the recommendations in the Guide for the Care and Use of Laboratory Animals of the National Institutes of Health and the American Veterinary Medical Association Guidelines on Euthanasia. The animal protocol was approved by the Institutional Animal Care and Use Committee at the University of Florida (study number 201104914).

### Virus-specific enzyme-linked immunosorbent assay

Serum and IC samples were tested for virus-specific IgG and IgA, respectively, by ELISA. For antigen, the ORF2+ORF3+3′ untranslated region of MNV-1 and MNV-3 genomes was cloned into pTriEx-1.1 Hygro for the purpose of baculovirus generation and expression of recombinant VP1+VP2 (rVP1/2) proteins in Sf9 insect cells. Expression was carried out by the Baculovirus/Monoclonal Antibody Facility at Baylor College of Medicine. Sf9 supernatants from recombinant baculovirus-infected cells were clarified by centrifugation at 2000× g for 30 min and analyzed by electron microscopy to determine whether virus-like particles (VLPs) were formed. Specifically, 10 µl of the clarified Sf9 supernatant was adsorbed on a 400-mesh nickel grid coated with formvar/carbon film (Electron Microscopy Science) for 1 min. The grids were washed once in PBS-EM (20.8 mM Na_2_PO_4_, 6.4 mM KH_2_PO_4_, 40 mM NaN_3_, and 300 mM NaCl, pH 7.2), fixed with 1% glutaraldehyde in PBS-EM for 1 min, washed in PBS-EM and dH_2_O twice, and stained with 0.5% uranyl acetate in H_2_O for 1 min. The excess liquid was gently wicked off and the grid was allowed to air dry. For immunogold labeling, the grids were incubated in blocking solution (3.0% BSA+0.1% NP40 in PBS-EM) for 30 min at room temperature followed by incubation with anti-MNV-1 VP1 antibody [40] in blocking solution at a 1∶2000 dilution for 30 min. The grids were washed three times with PBS-EM and incubated with gold conjugated anti-rabbit antibody (Ted Pella, Inc) for 30 min. The grids were then washed three times in PBS-EM, fixed for 5 min with McDowell Trumps fixative, washed two times with dH_2_O, stained with 0.5% uranyl acetate, and processed as described above. Samples were visualized on a Hitachi H7000 transmission electron microscope. For the ELISA, 250 ng of MNV-1 or MNV-3 rVP1/2 was dispensed into 96-well plates and incubated at room temperature overnight. After washing and blocking with 0.1% BSA, 1∶10 dilutions of serum samples or undiluted IC samples were added and plates were incubated at 37°C for 1 h. After washing, sheep anti-mouse IgG conjugated to horse radish peroxidase (HRP) was added to wells incubated with serum samples and goat anti-mouse IgA HRP was added to wells incubated with IC. Plates were incubated at 37°C for 45 min. After ABTS substrate addition, absorbance values were read at 415 nm using a Spectramax M2 plate reader. As a positive control for rVP1/2 integrity, we confirmed that MNV-1 and MNV-3 antigens were recognized equivalently by a polyclonal anti-MNV-1 capsid antibody (data not shown). To control for plate-to-plate variability, we tested serial dilutions of a positive control MNV-3 serum against both MNV-1 and MNV-3 rVP1/2 on each ELISA plate.

### Neutralization assays

Serum samples collected from B6 mice that were inoculated with mock inoculum or 10^4^ TCID_50_ units of either MNV-1 or MNV-3 for six weeks were incubated for 30 min at 56°C to inactivate complement. These samples were then serially diluted and each dilution was incubated with 3×10^4^ TCID_50_ units of homologous virus for 1 h at 37°C. The infectivity of each sample was determined by standard TCID_50_ assay. The endpoint of neutralization was determined as the highest dilution that reduced the viral titer by 50%.

### Passive transfers

Donor B6 mice were inoculated perorally with mock inoculum, 10^4^ TCID_50_ units of MNV-1, or 10^4^ TCID_50_ units of MNV-3. After six weeks, blood was collected by cardiac puncture, centrifuged in serum separator tubes, and incubated at 56°C for 30 min to inactivate complement. For passive transfer into recipient B6 and RAG1^−/−^ mice, the indicated volume of donor serum was injected intraperitoneally. One day later, recipient mice were challenged with 10^7^ TCID_50_ units of MNV-1 or MNV-3. At 1 dpi, indicated tissues were dissected for virus load determination.

### Adoptive transfers

Donor B6 mice were inoculated perorally with mock inoculum or 10^4^ TCID_50_ units of MNV-3. After six weeks, mice were boosted with mock inoculum or 10^7^ TCID_50_ units of MNV-3 for one day and spleens were dissected and single cell suspensions generated. CD4^+^ T cells were isolated from total splenocytes using negative selection magnetic beads (STEMCELL Technologies Inc.). The purity of CD4^+^ T cells was measured by flow cytometric analysis and determined to be >95% in each experiment. For adoptive transfer, 2×10^6^ CD4^+^ T cells collected from mock- or MNV-3-immunized mice were injected intraperitoneally into recipient RAG1^−/−^ mice. One day later, recipient mice were challenged with 10^7^ TCID_50_ units of MNV-3. At 1 dpi, indicated tissues were dissected for virus load determination.

### Virus growth curves

For infections, RAW 264.7 cells were infected at either MOI 0.05 or 5 and incubated for 1 h on ice. Cells were washed once with PBS to remove unbound virus and incubated at 37°C. For transfections, viral genomic RNA was purified from uncloned virus stocks with the RNeasy Mini Kit (Qiagen). RAW 264.7 or HEK-293T cells were transfected with the indicated quantity of RNA using Lipofectamine 2000 (Invitrogen) and incubated at 37°C. For all growth curves, culture supernatants were collected at the indicated time points and centrifuged at 10,000 rpm for 5 min. Virus titers were determined using TCID_50_ assay.

### Western blots

Infected or transfected cells were lysed in 1× Laemmli sample buffer. Proteins were separated by SDS-PAGE and transferred to polyvinylidene fluoride (PVDF) membranes. Polyclonal MNV-1-specific antibodies to protease-polymerase (ProPol) [54], VP1 [40], and VP2 [41] have been described; the latter was donated by Dr. Ian G. Goodfellow (University of Cambridge). Monospecific affinity-purified anti-VP2 antibody was produced by immunizing rabbits with the synthetic peptide C TQIQAQKDLTLMGQQFN (Pacific Immunology). The amino-terminal non-virally encoded cysteine residue (underlined) was included to allow for conjugation to a carrier protein. Mouse anti-actin was purchased from EMD Millipore and anti-His was purchased from Thermo Scientific. Blots were stripped using Restore PLUS Western Blot Stripping Buffer (Thermo Scientific) for the purpose of re-probing.

### Generation of ORF2-4 expression plasmids

All genes were amplified with primers (Table 1) containing restriction sites (underlined) from pSPMNV-1.CW3 and pSPMNV-3 to generate constructs for MNV-1 and MNV-3, respectively. The resultant PCR products generated by amplification with *PfuUltra* II (Aligent Technologies) were digested, gel-purified, and ligated into the pTriEx-1.1 Hygro vector (Novagen). The constructs were designated pTriEx-1ORF2, pTriEx-1ORF3, pTriEx-1ORF4, pTriEx-3ORF2, pTriEx-3ORF3 and pTriEx-3ORF4.

**Table 1:** Primers for amplifying MNV genes.

Primer Name	Primer Sequence	Restriction Site
ORF2-sense	5′-GCGCGATATCTAGGATGAGTGATGGC-3′	EcoRV
MNV1-ORF2-antisense	5′-GCGCCTCGAGTTATTGTTTGAGCATTCGG-3′	XhoI
MNV3-ORF2-antisense	5′-GCGCCTCGAGTTATTGTTTGAGTGTTCGG-3′	XhoI
MNV1-ORF3-sense	5′-GCGCCCATGGCTGGTGCTCTTTTCGGAGC-3′	*Nco*I
MNV3-ORF3-sense	5′-GCGCCCATGGCTGGTACTCTTTTCGGAGC-3′	NcoI
ORF3-antisense	5′-GCGCGCGGCCGCCTATGCCCTGCTACTCCCG-3′	NotI
ORF4-sense	5′-GCGCGATATCTGCGCAGCGCCAAAAGC-3′	EcoRV
MNV1-ORF4-antisense	5′-GCGCCTCGAGGGTAGACAAAATTG-3′	XhoI
MNV1-ORF4-antisense	5′-GCGCCTCGAGGGTATACAAAGTTG-3′	XhoI

### Cytokine analyses

To quantify cytokine transcripts, total RNA was extracted from infected cells with the RNeasy Mini Kit (Qiagen) and cDNA was synthesized using the ImProm-II Reverse Transcription System (Promega). Quantitative real-time PCR was carried out using an iCycler iQ (Bio-Rad) with SYBR Green Master Mix (Thermo Scientific). Primers for IFN-β, TNFa, and MCP-1 were purchased from Real Time Primers LLC. Primers for GAPDH were sense 5′-CATGGCCTTCCGTGTTCCTA-3′ and antisense 5′-CCTGCTTCACCACCTTCTTGAT-3′. Normalized cytokine levels were calculated using the comparative Ct method.

### IFN-β luciferase reporter assay

5×10^4^ HEK-293T cells were inoculated into wells of a 96-well plate and grown in DMEM with 10% FBS without antibiotics at 37°C. Following overnight incubation, each well was transfected with125 ng of total plasmid DNA including 10 ng of pβLUX in which the firefly luciferase gene is driven by the IFN-β promoter; 15 ng of pRL-SV40 in which renilla luciferase is driven by the constitutive SV40 promoter; 50 ng of pN-RIGI which expresses a constitutively active RIG-I; and 50 ng of test plasmid or filler pTriEx-1.1 Hygro. The pβLUX and pN-RIGI constructs were donated by Dr. Matthew B. Frieman (University of Maryland) and the pRL-SV40 construct was donated by Drs. William Klimstra and Kate Ryman (University of Pittsburgh). Transfections were performed with Lipofectamine 2000 (Invitrogen). At 24 h post-transfection, firefly and renilla luciferase levels were measured using the Dual-Glo Luciferase Assay System (Promega). Luminescence was measured using a ThermoScientific Appiskan plate reader. Data are presented as the ratio of firefly to renilla luciferase values.

### Flow cytometric analysis

RAW 264.7 cells were stimulated with 50 µg/ml of poly(I:C) (InvivoGen) or infected with recombinant MNV-1, MNV-3, MNV-1.3VP2, or MNV-3.1VP2 at MOI 5. For negative controls, PBS, mock inoculum, or UV-inactivated MNV-1 or MNV-3 was added to cells. Parental viruses were UV-inactivated by exposing them to 250,000 µJ cm^−2^ UV for 30 min on ice, as previously described [56], [57], and inactivation was confirmed by TCID_50_ assay. Cells were incubated at 37°C for the indicated amount of time and then stained with the following fluorescently conjugated antibodies in PBS containing 5% bovine serum albumin: anti-MHC class I, anti-MHC class II, anti-CD40, anti-CD80, or anti-CD103 (eBioscience) for 30 min at 4°C. Matched isotype controls were used for all antibodies. Flow cytometric analysis was performed on a FACSCalibur instrument (BD Biosciences) and data were analyzed using FCS Express 4 software.

### Translation termination-reinitiation assays

Individual or combinatorial changes were introduced into the previously reported MNV-1 TTR reporter plasmid [42] using the Quikchange II Site-Directed Mutagenesis Kit. Specific changes that were introduced are as follows: the 1-U/C construct contains a U to C mutation at positive 6644 of the MNV-1 genome; the 1-UG/CA construct contains two mutations from UG to CA at positions 6668 and 6669 of the MNV-1 genome; and WT-3 contains the 6644, 6668, and 6669 mutations, converting the MNV-1 TTR sequence into the wild-type MNV-3 sequence. Reporter plasmids were linearized with *HpaI* and capped run-off transcripts generated using T7 RNA polymerase as described [58]. Messenger RNAs were recovered by a single extraction with phenol/chloroform (1∶1 *v/v*) followed by ethanol precipitation. Remaining unincorporated nucleotides were removed by gel filtration through a NucAway spin column (Ambion). The eluate was concentrated by ethanol precipitation, the mRNA resuspended in water, checked for integrity by agarose gel electrophoresis and quantified by spectrophotometry. Messenger RNAs were then translated in Flexi-rabbit reticulocyte lysate (Flexi-RRL, Promega) programmed with 50 µg/ml template mRNA. Reactions were of 10 µl and composed of 60% (v/v) Flexi-RRL, 20 µM amino acids (lacking methionine), 500 µM MgOAc, 2 mM DTT, 5U RNAse inhibitor (RNAguard, GE Healthcare Life Sciences), 130 mM-160 mM KCl (optimised for each batch of Flexi-RRL) and 0.2 MBq [^35^S]-methionine. Reactions were incubated for 1 h at 30°C and stopped by the addition of an equal volume of 10 mM EDTA, 100 µg/ml RNase A followed by incubation at room temperature for 20 minutes. Samples were prepared for SDS-PAGE by the addition of 10 volumes of 2× Laemmli sample buffer and boiling for 3 minutes, and then resolved on 12% SDS-PAGE gels. The relative abundance of products on the gels was determined by direct measurement of [^35^S]methionine incorporation using a Packard Instant Imager 2024.

### Statistical analysis

All data analyses were performed using GraphPad Prism software. In all graphs, standard errors of mean were used to define error bars and *P* values were determined using unpaired two-tailed *t* tests. One asterisk represents *P* values of 0.01 to 0.05, two asterisks represent *P* values of 0.001 to 0.01, and three asterisks represent *P* values of less than 0.001.

## Supporting Information

Figure S1 **A monospecific anti-VP2 peptide antibody, but not a polyclonal anti-VP2 antibody, recognizes MNV-1 and MNV-3 VP2 equivalently.** **A**) HEK-293T cells were transfected with 0.2 µg of pTriEx-1ORF3 (1) or pTriEx-3ORF3 (3) and cell extracts prepared at the indicated times post-transfection (h post-tx). Western blots were carried out with the polyclonal anti-MNV-1 VP2 antibody, followed by stripping and re-probing with anti-His antibody. **B**) The amino acid sequences of MNV-1 and MNV-3 VP2 proteins are shown, with differences underlined in the MNV-3 protein sequence. The conserved peptide indicated in blue was used to generate a monospecific anti-VP2 antibody. **C**) Cell extracts from RAW 264.7 cells infected with MNV-1 or MNV-3 at MOI 5 were blotted with either the polyclonal or the monospecific anti-VP2 antibodies. **D**) Translation termination-reinitiation (TTR) assays were carried out as described in the Methods. The products were resolved by 12% SDS-PAGE and visualised by autoradiography. Bands of the sizes expected for rlucVP1 ORF (42 kDa) and VP2flucORF (64 kDa) are indicated. The numbers underneath each band denote the relative reinitiation frequency in comparison to WT-1 set at 100.
(TIF)
