# Supplementary figures and images for: Identification of Immune and Viral Correlates of Norovirus Protective Immunity through Comparative Study of Intra-Cluster Norovirus Strains
Source: PLoS Pathog. 2013 Sep 5;9(9):e1003592. doi: 10.1371/journal.ppat.1003592 (PMC3764223; doi:10.1371/journal.ppat.1003592)

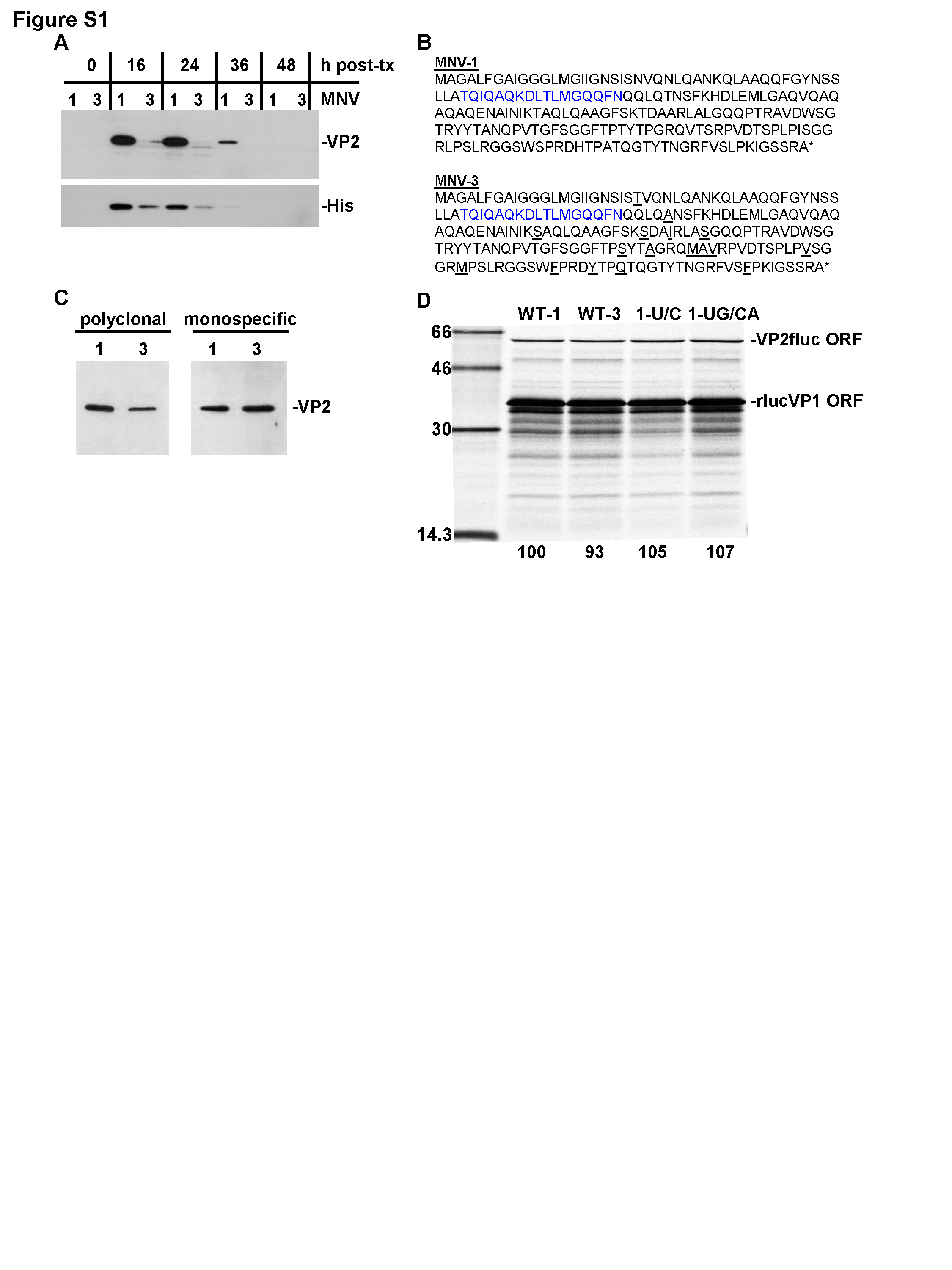

Supplement: Figure S1 — A monospecific anti-VP2 peptide antibody, but not a polyclonal anti-VP2 antibody, recognizes MNV-1 and MNV-3 VP2 equivalently. A) HEK-293T cells were transfected with 0.2 µg of pTriEx-1ORF3 (1) or pTriEx-3ORF3 (3) and cell extracts prepared at the indicated times post-transfection (h post-tx). Western blots were carried out with the polyclonal anti-MNV-1 VP2 antibody, followed by stripping and re-probing with anti-His antibody. B) The amino acid sequences of MNV-1 and MNV-3 VP2 proteins are shown, with differences underlined in the MNV-3 protein sequence. The conserved peptide indicated in blue was used to generate a monospecific anti-VP2 antibody. C) Cell extracts from RAW 264.7 cells infected with MNV-1 or MNV-3 at MOI 5 were blotted with either the polyclonal or the monospecific anti-VP2 antibodies. D) Translation termination-reinitiation (TTR) assays were carried out as described in the Methods. The products were resolved by 12% SDS-PAGE and visualised by autoradiography. Bands of the sizes expected for rlucVP1 ORF (42 kDa) and VP2flucORF (64 kDa) are indicated. The numbers underneath each band denote the relative reinitiation frequency in comparison to WT-1 set at 100. (TIF) [file ppat.1003592.s001.tif]
